# Synthesis of cationic dibenzosemibullvalene-based phase-transfer catalysts by di-π-methane rearrangements of pyrrolinium-annelated dibenzobarrelene derivatives

**DOI:** 10.3762/bjoc.7.17

**Published:** 2011-01-26

**Authors:** Heiko Ihmels, Jia Luo

**Affiliations:** 1 Organic Chemistry II, University of Siegen, Adolf-Reichwein-Str. 2, D-57068 Siegen, Germany

**Keywords:** di-π-methane rearrangement, dibenzobarrelenes, dibenzosemibullvalenes, phase-transfer catalysis, photochemistry, polycylic compounds

## Abstract

Dibenzobarrelene derivatives, that are annelated with a pyrrolinium unit [*N*,*N*-dialkyl-3,4-(9',10'-dihydro-9',10'-anthraceno-3-pyrrolinium) derivatives], undergo a photo-induced di-π-methane rearrangement upon triplet sensitization to give the corresponding cationic dibenzosemibullvalene derivatives [*N,N*-dialkyl-3,4-{8c,8e-(4b,8b-dihydrodibenzo[*a,f*]cyclopropa[*cd*]pentaleno)}pyrrolidinium derivatives]. Whereas the covalent attachment of a benzophenone functionality to the pyrrolinium nitrogen atom did not result in an internal triplet sensitization, the introduction of a benzophenone unit as part of the counter ion enables the di-π-methane rearrangement of the dibenzobarrelene derivative in the solid-state. Preliminary experiments indicate that a cationic pyrrolidinium-annelated dibenzosemibullvalene may act as phase-transfer catalyst in alkylation reactions.

## Introduction

**Scheme 1:**
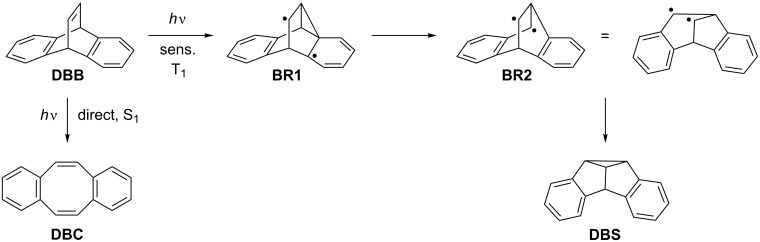
Photorearrangements of dibenzobarrelene (**DBB**).

The di-π-methane (DPM) rearrangement is among the most thoroughly investigated organic photoreactions [1–2]. Since the discovery of the DPM rearrangement of dibenzobarrelene [3], extensive studies have been carried out to assess the mechanistic aspects of this reaction and to explore their application in synthetic organic chemistry [1–5]. Along these lines, the DPM rearrangement of dibenzobarrelene (**DBB**), also termed dibenzobicyclo[2.2.2]octatriene, and its derivatives has been investigated in detail and has provided significant insights into the mechanism of the DPM rearrangement [6–7]. The DPM rearrangement of **DBB** proceeds according to the mechanism shown in Scheme 1 through biradical intermediates **BR1** and **BR2** and leads to the formation of the corresponding dibenzosemibullvalene (**DBS**) [6–7]. Notably, the photoreactivity of the dibenzobarrelene system is multiplicity-dependent: Upon photoinduced triplet sensitization in the presence of an appropriate sensitizer, such as acetone or benzophenone, dibenzobarrelene (**DBB**) rearranges to dibenzosemibullvalene (**DBS**), whereas the direct excitation leads to dibenzocyclooctene (**DBC**) through the singlet excited-state (Scheme 1) [4–5,7]. The DPM photorearrangement of dibenzobarrelene and its derivatives is a synthetically useful reaction, because it allows the preparation of polycyclic structures which are relatively difficult to obtain by ground-state transformations [8].

**Figure 1:**
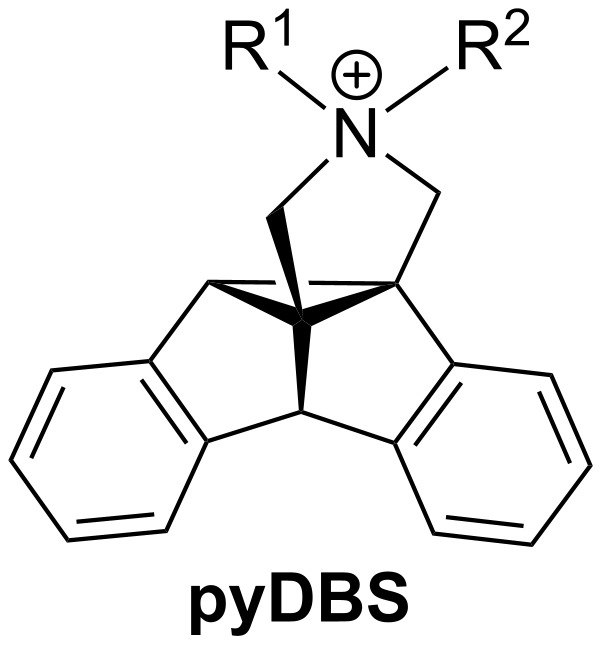
General structure of pyrrolidinium-annelated dibenzosemibullvalenes (**pyDBS)**.

During our attempts to develop novel ammonium-based phase-transfer catalysts [9–11] that are embedded within a rigid structure, we noticed that the complex dibenzosemibullvalene structure may constitute a reasonable starting point. We proposed that the general structure **pyDBS** (Figure 1), as established by the annelation of a pyrrolidinium unit to the dibenzosemibullvalene structure [12], represents an amphiphilic tetraalkylammonium derivative that exhibits three different benzene-containing concave sites to which an organic substrate may associate by attractive van der Waals interactions or π-stacking. Moreover, additional functionalities, e.g., hydroxy or amino groups, may be attached to the benzene rings of the semibullvalene structure to enable additional hydrogen bonding with the substrate. Also, the benzene rings may be further annelated with additional aromatic units to increase the potential π-stacking area [13]. Most notably, dibenzosemibullvalenes are chiral compounds, so that the propensity of an enantiopure **pyDBS** derivative to act as phase-transfer catalyst in stereoselective reactions may be considered in long-term studies. Herein we demonstrate that the pyrrolidinium-annelated dibenzosemibullvalene structure is indeed available via the DPM rearrangement of appropriately substituted dibenzobarrelene derivatives and that such a compound may act as phase-transfer catalyst in alkylation reactions.

Note that according to the IUPAC rules the compounds presented in the following should be classified as pyrrolidinium or pyrrolinium derivatives because of the higher priority of the cationic heterocycles; however, for clarity and to keep the focus on the photochemical reaction we chose to name these compounds dibenzobarrelene and dibenzosemibullvalene derivatives throughout the text.

## Results and Discussion

**Scheme 2:**
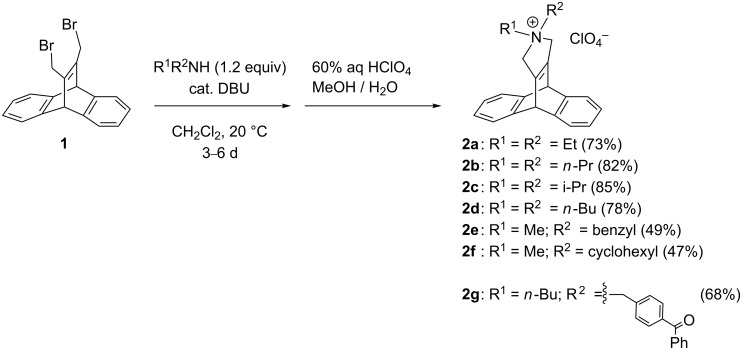
Synthesis of dibenzobarrelene derivatives **2a**–**g**.

The cationic dibenzobarrelene derivatives **2a**–**g** were synthesized by the base-catalyzed reaction between the dibromomethyl-substituted dibenzobarrelene derivative **1** [14] with selected secondary amines (Scheme 2). The reaction was initially performed with a slight excess of the amine and DBU as a base in dichloromethane at room temperature, but under these conditions the products could not be completely separated from the remaining amine or the DBU catalyst, even by column chromatography. Nevertheless, the products were available in reasonable yields (47–85%) when the reaction was performed with polymer-bound DBU as catalyst (**2a**–**f**: 0.1 equiv; **2g**: 0.5 equiv) or when the quaternary ammonium derivatives were precipitated from aqueous solutions by the addition of perchloric acid to the reaction mixture and, if necessary, subsequent column chromatography. All products were identified and fully characterized by NMR spectroscopy, mass spectrometry and elemental analyses. In general, the perchlorate salts of **2a**–**g** have good solubility in aprotic polar solvents, especially in acetone and acetonitrile. To confer water-solubility on the cationic dibenzobarrelene derivatives **2b** and **2d**, the perchlorate salts were converted quantitatively to chlorides by ion exchange with chloride ions on an ion exchange resin.

**Scheme 3:**
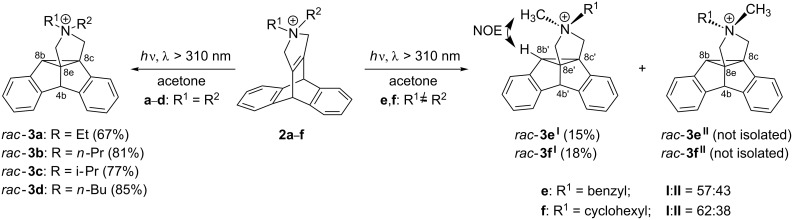
Di-π-methane rearrangements of dibenzobarrelene derivatives **2a**–**f** (counter ions omitted for clarity).

The photolysis of the cationic dibenzobarrelene derivatives **2a**–**d**, which bear two identical *N*-alkyl substituents, was carried out in acetone solutions (λ > 310 nm), and the conversion of the dibenzobarrelene was monitored by ^1^H NMR spectroscopy. The dibenzosemibullvalene derivatives **3a**–**d** were isolated by crystallization directly from the photolysate in 67–85% yield. The photoproducts were identified and characterized by ^1^H NMR and ^13^C NMR spectroscopy, mass spectrometry, and elemental analyses. The dibenzosemibullvalene structure of the photoproducts **3a**–**d** was confirmed by ^1^H NMR spectroscopy, specifically by the characteristic singlets of the protons at the dibenzylic position at C4b (ca. 4.9 ppm) and of the cyclopropyl protons at C8b (ca. 4.0 ppm) (Scheme 3). Direct irradiation (quartz filter, λ > 254 nm) of the dibenzobarrelene derivatives **2a**–**d** resulted in complex reaction mixtures in which the characteristic ^1^H NMR signals of dibenzosemibullvalene or dibenzocyclooctene products were not detected. Presumably, the photo-induced cleavage of the C–N bonds, i.e., a well-known photo-fragmentation of quaternary ammonium salts to give highly reactive radical intermediates [15–16], is a significant side-reaction during the direct photolysis of compounds **2a**–**d**, leading to the formation of complex reaction mixtures. The triplet-sensitized photoreaction of the dibenzobarrelene derivatives **2e**,**f**, which carry two different alkyl substituents at the quaternary pyrrolidinium, gave two isomeric dibenzosemibullvalene products **3e^I^**,**3f^I^** and **3e^II^**,**3f^II^** , in a ratio of approximately 60:40 as determined by ^1^H NMR spectroscopic analysis of the reaction mixture (Scheme 3). The comparison was based on the two characteristic singlets of the protons at C4b and C8b of the dibenzosemibullvalene ring (**3e^I^**: 4.30, 4.75; **3e^II^**: 4.17, 4.91; **3f^I^**: 4.31, 5.05 ppm; **3f^II^**: 4.20, 5.02, in CDCl_3_). After column chromatography and subsequent recrystallization, the major photoproducts **3e^I^** and **3f^I^** were isolated in low yields (15% and 18%). The relative configuration of the ammonium functionality was deduced from NOE experiments. Specifically, the close proximity between the methyl group and the methine proton (8b’-H) of the cyclopropane ring gave rise to an NOE in **3e^I^** and **3f^I^** (Scheme 3). The structure of the minor products **3e^II^** and **3f^II^**, although not isolated, were determined by ^1^H NMR spectroscopic analyses of the photolysates. In these cases, the dibenzosemibullvalene structure was indicated by the characteristic ^1^H NMR spectroscopic shifts of the 4b-H and 8b-H protons.

**Scheme 4:**
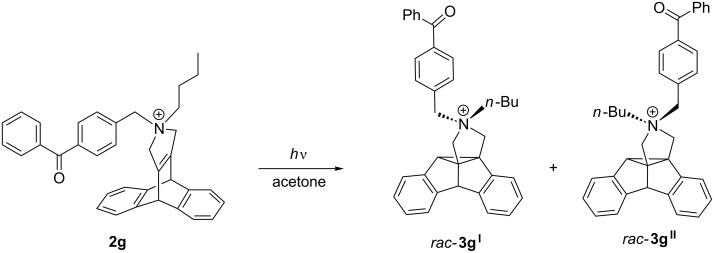
Di-π-methane rearrangement of dibenzobarrelene derivative **2g**.

It has been demonstrated with several examples that solid-state photoreactions are an excellent tool to induce highly selective di-π-methane rearrangements of dibenzobarrelene derivatives [17], because the constrained medium within the crystal lattice allows only limited molecular movement, so that the reaction pathway with the least required molecular motion is preferred. Accordingly, the solid-state photoreactivity of the dibenzobarrelene derivatives **2a**–**f** was investigated; but unfortunately, all tested derivatives, either as chloride or as perchlorate salts, were photoinert in the crystalline state. Since one of the possible reasons for this photoinertness may be the lack of proper sensitization, the dibenzobarrelene derivative **2g** was prepared with a triplet-sensitizing functionality attached, namely the benzophenone unit. Although in acetone the dibenzobarrelene **2g** underwent a DPM rearrangement with full conversion (Scheme 4), the irradiation of **2g** in water or acetonitrile solution in the absence of an external sensitizer only induced a relatively low conversion of **2g**. Thus, irradiation of **2g** in acetonitrile for 12 h led to ca. 10% conversion with ca. 60% dibenzosemibullvalenes formed. At the same time, several unidentified byproducts were formed in significant amounts. The analysis of the photolysate by ^1^H NMR spectroscopy revealed that independent of the solvent, the two semibullvalenes **3g^I^** and **3g^II^** were formed in a 45:55 ratio; however, attempts to separate the two regioisomers by chromatography were unsuccessful (Scheme 4). Derivative **2g** was also photoinert in the solid-state.

The lack of internal photosensitization of derivative **2g** resembles the behavior of the hydrochloride salt of a cationic ammoniummethyl-substituted dibenzobarrelene derivative [18] which is also photoinert in the presence of benzophenone and even acetone. It may be assumed that attachment of the benzophenone unit to the cationic dibenzobarrelene structure has an influence on the intersystem-crossing (ISC) rate or the triplet energy of the carbonyl functionality leading to insufficient sensitization. In addition, competing deactivation pathways of the excited ketone may be considered, such as rotation about the C_ar_–CH_2_ group or reaction with the chloride counter ion. However, further attempts to clarify this aspect or to optimize the conditions for sensitization were not made, because the photoreaction of **2g** upon external sensitization by acetone showed that the products of the DPM rearrangement of **2g** are formed only slowly in relatively low yield and cannot be separated.

**Scheme 5:**
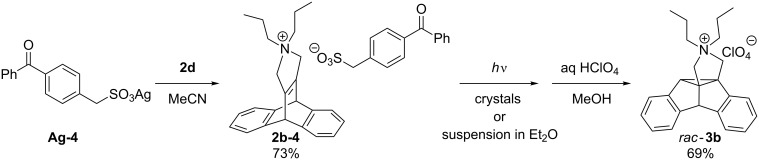
Synthesis and solid-state photoreactivity of the sulfonate salt **2b-4**.

Since the covalent attachment of a sensitizer unit to the dibenzobarrelene chromophore did not induce the desired triplet sensitization, the ionic auxiliary strategy [19] was applied to achieve triplet-sensitization in the solid-state, i.e., the sensitizer was introduced as counter anion. For this purpose, an anionic derivative of benzophenone was prepared and associated with the ammonium functionality of the dibenzobarrelene **2b** by anion metathesis (Scheme 5). The known sulfonic acid **4** [20] was transformed to the corresponding silver salt **Ag-4** by reaction with Ag_2_O, and subsequent ion metathesis upon treatment with **2b-Cl** in acetonitrile gave the salt **2b-4** in 73% yield. Irradiation of ground crystals of the salt **2b-4** for 2 h induced a DPM rearrangement to the dibenzosemibullvalene derivative **3b** with full conversion and without any detectable byproducts as determined by ^1^H NMR spectroscopy. A solid-state photoreaction was also performed by the irradiation of a suspension of crystalline **2b-4** in diethyl ether. The solid products were dissolved and precipitated as perchlorate salts. Although the dibenzosemibullvalene **3b** was formed (68%), ^1^H NMR spectroscopic analysis of the photolysate indicated the additional formation of significant amounts of byproducts (ca. 15%). These results indicate that, in principle, the internal sensitization of the DPM rearrangement of the cationic dibenzobarrelene derivatives may be achieved in a solid-state reaction with a sensitizing counter ion; however, the sensitization by acetone solvent appears to be more practical as it allows the handling of larger scales.

**Scheme 6:**
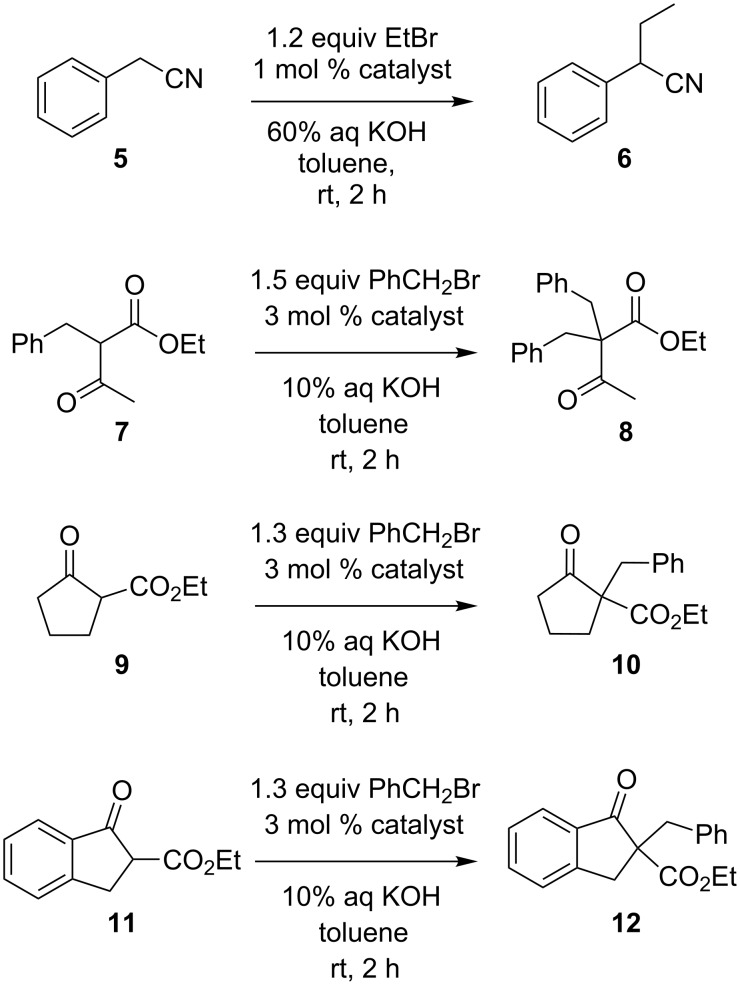
Phase-transfer catalyzed alkylation reactions (see Table 1 for details).

The general catalytic ability of the dibenzosemibullvalene salt **3d** in phase-transfer catalyzed nucleophilic substitution reactions was investigated. Compounds **5** [21], **7**, **9**, and **11** were chosen as substrates in alkylation reactions under phase-transfer conditions, and the catalytic activity of **3d** was compared with a tetrabutylammonium salt (Scheme 6, Table 1; TBAB = tetrabutylammonium bromide; TBAC = tetrabutylammonium chloride.). All four reactions under investigation were significantly accelerated in the presence of substoichiometric amounts of the quaternary ammonium catalysts. Notably, under identical conditions the dibenzosemibullvalene salt **3d** induced higher conversions in the phase-transfer reactions than the tetrabutylammonium salts, except for the alkylation of compound **5**, in which 92% conversion was achieved using TBAB, whereas under identical conditions a conversion of only 32% was obtained with dibenzosemibullvalene **3d** as the catalyst. In contrast, the alkylation of the β-oxoester **7** in the presence of **3d** gave the product in 82% conversion after 2 h at room temperature, whereas with TBAC as catalyst the conversion was only 36% under identical conditions. The dibenzosemibullvalene salt **3d** was also found to be more efficient in the alkylation of cyclic β-oxoesters **9** and **11**, as compared with the TBAC catalyst (Table 1). Interestingly, the alkylation of the indanone derivative **11** is less efficient in the presence of TBAC as compared with the non-aromatic cyclopentanone derivative **9**, whereas in the presence of dibenzosemibullvalene **3d** both substrates are alkylated with comparable efficiency.

**Table 1:** Phase-transfer catalyzed alkylation reactions according to Scheme 6.

Substrate	Catalyst^a^	Conv.^b^ / %

**5**	–	5^c^
**5**	TBAB	92^c^
**5**	**3d**	32^c^
**7**	–	2
**7**	TBAC	36
**7**	**3d**	82
**9**	–	5
**9**	TBAC	81
**9**	**3d**	95
**11**	–	4
**11**	TBAC	49
**11**	**3d**	>97

^a^TBAB = tetrabutylammonium bromide; TBAC = tetrabutylammonium chloride. ^b^Conversion determined by ^1^H NMR spectroscopic analysis of the reaction mixture, relative to 2,7-dimethyl naphthalene as internal standard; estimated error: ±3% of the given value. ^c^Determined by GC analysis, relative to ethyl acetate as internal standard.

## Conclusion

In summary, it was shown that cationic pyrrolinium-annelated dibenzosemibullvalene derivatives are accessable by the photoinduced di-π-methane rearrangement of appropriately substituted dibenzobarrelene substrates. With one representative example, it was demonstrated that these compounds may act as phase-transfer catalysts in alkylation reactions with comparable or even better performance than the commonly employed tetrabutylammonium salts. It is therefore concluded that this class of chiral compounds may be used as a promising platform for the development of versatile phase-transfer catalysts.

## Experimental

**General remarks:** NMR spectra were recorded on a Bruker Avance 400 (^1^H NMR: 400 MHz; ^13^C NMR: 100 MHz) and a Varian NMR system 600 (^1^H NMR: 600 MHz; ^13^C NMR: 150 MHz). ^1^H NMR chemical shifts are given relative to δ_TMS_ = 0.00 ppm, and ^13^C NMR chemical shifts refer to either the TMS signal (δ_TMS_ = 0.00 ppm) or the solvent signals [CDCl_3_: 77.0 ppm; CD_3_OD: 49.0 ppm; (CD_3_)_2_CO: 29.8 ppm, CD_3_CN: 118.2 ppm, (CD_3_)_2_SO: 39.5 ppm]. Melting points were determined with a Büchi 510K and are uncorrected. Mass spectra were recorded on a Hewlett-Packard HP 5968 (EI) and a Finnigan LCQ Deca instrument (ESI). Elemental analyses were performed on a KEKA-tech EuroEA combustion analyzer by Mr. H. Bodenstedt, Organic Chemistry I, University of Siegen. TLC analyses were performed on silica-gel sheets (Macherey-Nagel Polygram Sil G/UV_254_). Unless otherwise noted, commercially available chemicals were reagent grade and used without further purification. Polymer-bound DBU (polystyrene cross-linked with 1% divinylbenzene; loading: 1.15 mmol/g) was obtained from Aldrich. Anhydrous THF and diethyl ether were obtained by distillation from sodium wire, and anhydrous CH_2_Cl_2_ was distilled from calcium hydride. Distilled water was used for all the synthetic reactions performed. Preparative column chromatography was performed on MN Silica Gel 60 M (particle size 0.04–0.063 mm, 230–440 mesh).

### Synthesis of dibenzobarrelene derivatives

**General procedure for the preparation of *N*,*N*-dialkyl-3,4-(9',10'-dihydro-9',10'-anthraceno-3-pyrrolinium derivatives (GP-1)**: A mixture of 11,12-bis(bromomethyl)-9,10-dihydro-9,10-ethenoanthracene (**1**, 1.0–5.0 mmol), the corresponding secondary amine (1.2 equiv) and catalytic amount of DBU (0.1 equiv or otherwise explicitly specified) in dichloromethane (10 ml/mmol of **1**) was stirred at room temperature for 3–6 days until TLC analysis indicated the full conversion of **1**. The solvent was removed in vacuo and the residue re-dissolved in methanol (10–25 ml depending on the scale and solubility); if necessary, gentle heating was applied to dissolve the residue. Aqueous perchloric acid (60%, 1–3 ml) was added to the mixture. The perchlorate salt of the ammonium-dibenzobarrelene derivative precipitated spontaneously, or upon subsequent addition of a few drops of water. The solid was collected by filtration, washed with cold methanol and recrystallized from acetone. In those cases when no solid product precipitated from the methanol solution, water was added and the mixture extracted with dichloromethane. The organic layer was concentrated and the product purified by column chromatography (SiO_2_; 3–5% MeOH in dichloromethane).

The corresponding chloride salts were prepared by dissolving the perchlorate salt in MeCN and passing the solution through an ion-exchange column (DOWEX 1 x 8, Cl^−^ form). After removal of the solvent in vacuo, the residue was crystallized from Et_2_O/MeOH.

***N*,*N*-Di-*n*-butyl-3,4-(9',10'-dihydro-9',10'-anthraceno)-3-pyrrolinium perchlorate** (**2d**): Prepared from dibenzobarrelene **1** (1.60 g, 4.08 mmol) according to **GP-1**, yield 1.46 g (3.18 mmol, 78%), colorless prisms, mp 173–174 °C. ^1^H NMR [400 MHz, (CD_3_)_2_CO]: δ = 0.75 (t, *J* = 7 Hz, 6H, CH_3_), 1.20–1.26 (m, 4H, C*H_2_*CH_3_), 1.42–1.46 (m, 4H, C*H_2_*CH_2_CH_3_), 3.52–3.56 (m, 4H, NC*H_2_*CH_2_), 4.72 (s, 4H, C=CC*H_2_*N), 5.39 (s, 2H, C*H*), 6.99, 7.39 (AA’BB’-system, 8H, CH_ar_). ^13^C NMR [100 MHz, (CD_3_)_2_CO]: δ = 14.1 (CH_3_), 20.7 (CH_2_), 26.5 (CH_2_), 49.8 (CH_2_), 65.6 (CH_2_), 69.0 (CH), 124.8 (CH_ar_), 126.1 (CH_ar_), 144.6 (C_q_), 146.9 (C_q_). UV (CH_2_Cl_2_): λ_max_ (log ε) = 273 (4.00), 280 (4.17). MS (ESI^+^): *m/z* (%) = 358 (100). Anal. Calcd for C_26_H_32_ClNO_4_ (458.0): C, 68.18; H, 7.04; N, 3.06. Found: C, 68.22; H, 7.12; N, 3.05.

***N*,*N*-Di-*n*-butyl-3,4-(9',10'-dihydro-9',10'-anthraceno-3-pyrrolinium chloride** (**2d-Cl**): Obtained quantitatively (1.37 g, 3.47 mmol) as a white powder by ion exchange of the perchlorate **2d** (1.59 g, 3.47 mmol; eluent: acetonitrile; DOWEX resin). mp 194–195 °C. ^1^H NMR (400 MHz, CD_3_CN): δ = 0.78 (t, *J* = 7 Hz, 6H, CH_3_), 1.16–1.34 (m, 8H, C*H_2_*C*H_2_*CH_3_), 3.28–3.30 (m, 4H, NC*H_2_*CH_2_, overlapped with the solvent signal), 4.48 (s, 4H, C=CC*H_2_*N), 5.12 (s, 2H, C**H**), 6.99, 7.35 (AA’BB’-system, 8H, CH_ar_). ^13^C NMR (100 MHz, CD_3_CN): δ = 13.9 (CH_3_), 20.8 (CH_2_), 26.5 (CH_2_), 50.0 (CH_2_), 65.7 (CH_2_), 68.7 (CH), 126.2 (CH_ar_), 126.7 (CH_ar_), 144.6 (C_q_), 147.0 (C_q_).

### Synthesis of dibenzosemibullvalene derivatives

***General procedure for the photolysis in solution *(GP-2)**: Solutions of the substrates (10^−2^ to 10^−3^ mol/l) were placed in a 200 ml Duran flask (acetone) or a quartz test tube (other solvents), and argon gas was bubbled through the solution for at least 20 min. The solution was stirred and irradiated for 4–15 h until the starting material was fully converted, as determined by TLC or ^1^H NMR spectroscopic analysis. After evaporation of the solvent in vacuo, the photolysate was analyzed by ^1^H NMR spectroscopy, or, in preparative experiments, isolated by recrystallization or column chromatography to obtain the photoproduct.

***rac-N,N*-Di-*n*-butyl-4b’,8b’,8c’,8e’-dibenzo[*a*,*f*]cyclopropa[*cd*]pentaleno-pyrrolidinium perchlorate** (**3d**): Prepared from **2d** (50.0 mg, 0.11 mmol) according to **GP-2** in acetone solution as colorless prisms (42.0 mg, 0.09 mmol, 85%), mp 176–178 °C. ^1^H NMR (400 MHz, CD_3_CN): δ 0.61 (t, *J* = 7 Hz, 3H, CH_3_), 0.74 (t, *J* = 7 Hz, 3H, CH_3_), 0.85–0.94 (m, 1H, CH_2_), 0.99–1.07 (m, 1H, CH_2_), 1.16–1.33 (m, 4H, CH_2_), 1.33–1.39 (m, 2H, CH_2_), 2.74–2.82 (m, 1H, CH_2_), 2.88–2.95 (m, 2H, CH_2_), 3.48 (d, *J* = 13 Hz, 1H, NC*H*HC), 3.73 (d, *J* = 13 Hz, 1H, NC*H*HC), 4.04 (s, 1 H, CH), 4.24 (d, *J* = 13 Hz, 1H, NCH*H*C), 4.57 (d, *J* = 13 Hz, 1H, NCH*H*C), 4.91 (s, 1H, CH), 6.97–7.01 (m, 2H, CH_ar_), 7.09–7.17 (m, 4H, CH_ar_), 7.34–7.37 (m, 1H, CH_ar_), 7.39–7.41 (m, 1 H, CH_ar_), 7.38–7.40 (m, 1H, CH_ar_). ^13^C NMR (100 MHz, CD_3_CN): δ 13.0 (CH_3_), 13.6 (CH_3_), 20.1 (CH_2_), 20.3 (CH_2_), 24.8 (CH_2_), 25.8 (CH_2_), 55.5 (CH_2_), 55.7 (CH_2_), 59.8 (CH_2_), 60.0 (CH_2_), 61.9 (C_q_), 67.3 (CH), 69.4 (C_q_), 70.1 (CH), 122.5 (CH_ar_), 123.3 (CH_ar_), 125.8 (CH_ar_), 125.9 (CH_ar_), 127.7 (CH_ar_), 128.3 (CH_ar_), 128.4 (CH_ar_), 128.9 (CH_ar_), 137.0 (C_q_), 137.1 (C_q_), 152.0 (C_q_), 154.5 (C_q_). MS (ESI^+^): *m/z* (%) = 358 (100). Anal. Calcd for C_26_H_32_ClNO_4_ (458.0): C, 68.18; H, 7.04; N, 3.06. Found: C, 68.22; H, 7.14; N, 3.06.

### Phase-transfer catalyzed (PTC) alkylation reactions

**a) Analysis with gas chromatography**: A biphasic mixture of the quaternary ammonium catalyst **3d** or TBAC (0.05 mmol), phenylacetonitrile (**5**, 506 mg, 4.32 mmol) and bromoethane (808 mg, 4.75 mmol) in toluene (0.85 ml) and aqueous KOH solution (4 ml, 60%) was stirred for 2 h at 20 °C. Water (10 ml) and toluene (10 ml) were added and the mixture was thoroughly shaken. After phase separation, the organic layer was analyzed by gas chromatography.

*Stationary Phase*: HP-5 PhMe-Silica, crosslinked 5%, 25 m; *Mobile Phase*: Argon gas; *Flow Pressure*: 30 kPa; *Temperature*: Oven 220 °C, Injector 250 °C, Detector 250 °C; *Retention* time: PhCH_2_CN 9.6 min; PhCH(CH_2_CH_3_)CN 10.6 min.

Three PTC reactions were performed in parallel with a) no catalyst, b) TBAB as the catalyst, or c) **3d** as the catalyst.

**NMR-spectroscopically monitored PTC reactions**: A biphasic mixture of the quaternary ammonium catalyst (0.03 mmol), the substrate (0.10 mmol), benzylchloride (1.25–1.50 equiv) and 2,7-dimethylnaphthalene (as internal standard, 0.025 mmol) in toluene (5 ml) and aqueous KOH solution (5 ml, 10%) was stirred for 2 h at 20 °C. At the end of reaction, water (10 ml) and toluene (10 ml) were added and the mixture was thoroughly shaken. The separated organic phase was analyzed by ^1^H NMR spectroscopy, and the conversion calculated by comparison with the signal of the methyl protons of 2,7-dimethylnaphthalene (δ = 2.49 ppm, in CDCl_3_).

Three reactions were run in parallel with a) no catalyst, b) TBAC as the catalyst or c) **3d** as the catalyst.

## Supporting Information

File 1 Experimental procedures, characterization data and copies of ^1^H NMR and ^13^C NMR spectra of compounds **2a**–**g** and **3a**–**f**.
