# Supplementary material for: Synthesis of cationic dibenzosemibullvalene-based phase-transfer catalysts by di-π-methane rearrangements of pyrrolinium-annelated dibenzobarrelene derivatives
Source: Beilstein J Org Chem. 2011 Jan 26;7:119–26. doi: 10.3762/bjoc.7.17 (PMC3063005; doi:10.3762/bjoc.7.17)

Supporting Information

for

Synthesis of cationic dibenzosemibullvalene-based phase-transfer catalysts by di-π-methane rearrangements of pyrrolinium-annelated dibenzobarrelene derivatives

Heiko Ihmels* and Jia Luo

Address: Organic Chemistry II, University of Siegen, Adolf-Reichwein-Str. 2, D-57068 Siegen, Germany

Email: Heiko Ihmels - ihmels@chemie.uni-siegen.de

* Corresponding author

Experimental procedures, characterization data and copies of ^{1}H NMR and ^{13}C NMR spectra of compounds 2a–g and 3a–f.

Experimental

General remarks: NMR spectra were recorded on a Bruker Avance 400 (^{1}H NMR: 400 MHz; ^{13}C NMR: 100 MHz) and a Varian NMR system 600 (^{1}H NMR: 600 MHz; ^{13}C NMR: 150 MHz). ^{1}H NMR chemical shifts are given relative to δ_{TMS} = 0.00 ppm, and ^{13}C NMR chemical shifts refer to either the TMS signal (δ_{TMS} = 0.00 ppm) or the solvent signals [$CDCl_3$: 77.0 ppm; CD_3OD: 49.0 ppm; $(CD_3)_2CO$: 29.8 ppm, CD_3CN: 118.2 ppm, $(CD_3)_2SO$: 39.5 ppm]. Melting points were determined with a Büchi 510K and are uncorrected. Mass spectra were recorded on a Hewlett-Packard HP 5968 (EI) and a Finnigan LCQ Deca instrument (ESI). Elemental analyses were performed on a KEKA-tech EuroEA combustion analyzer by Mr. H. Bodenstedt, Organic Chemistry I, University of Siegen. TLC analyses were performed on silica-gel sheets (Macherey-Nagel Polygram Sil G/UV_{254}). Unless otherwise mentioned, commercially available chemicals were reagent grade and used without further purification.
Polymer-bound DBU (polystyrene crosslinked with 1% divinylbenzene; loading: 1.15 mmol/g) was obtained from Aldrich. Anhydrous THF and diethyl ether were obtained by distillation from sodium wire, and anhydrous CH_2Cl_2 was distilled from calcium hydride. Distilled water was used for all the synthetic reactions performed.
Preparative column chromatography was performed on MN Silica Gel 60 M (particle size 0.04–0.063 mm, 230–440 mesh).
Irradiations were performed with a TQ150 medium-pressure mercury lamp (Heraeus, UV-Consulting Peschl), which was placed inside a quartz cooling tube. The sample container was placed ca. 10–15 cm in front of the lamp.

Synthesis of dibenzobarrelene derivatives

General procedure for the preparation of *N*,*N*-dialkyl-3,4-(9',10'-dihydro-9',10'-anthraceno-3-pyrrolinium derivatives (GP-1): A mixture of 11,12-bis(bromomethyl)-9,10-dihydro-9,10-ethenoanthracene (**1**, 1.0–5.0 mmol), the corresponding secondary amine (1.2 equiv) and catalytic amount of DBU (0.1 equiv or otherwise explicitly specified) in dichloromethane (10 ml/mmol of **1**) was stirred at room temperature for 3–6 days until TLC analysis indicated the full conversion of **1**. The solvent was removed in vacuo and the residue re-dissolved in methanol (10–25 ml depending on the scale and solubility); if necessary, gentle heating was applied to dissolve the residue. Aqueous perchloric acid (60%, 1–3 ml) was added to the mixture. The perchlorate salt of the ammonium-dibenzobarrelene derivative precipitated spontaneously, or upon subsequent addition of a few drops of water. The solid was collected by filtration, washed with cold methanol and recrystallized from acetone. In those cases when no solid product precipitated from the methanol solution, water was added and the mixture extracted with dichloromethane. The organic layer was concentrated and the product purified by column chromatography (SiO_2; 3–5% MeOH in dichloromethane).

The corresponding chloride salts were prepared by dissolving the perchlorate salt in MeCN and passing the solution through an ion-exchange column (DOWEX 1 x 8, Cl^- form). After removal of the solvent in vacuo, the residue was crystallized from Et_2O/MeOH.

***N*,*N*-Diethyl-3,4-(9',10'-dihydro-9',10'-anthraceno)-3-pyrrolinium perchlorate** (**2a**): Prepared from dibenzobarrelene **1** (0.98 g, 2.49 mmol) according to **GP-1**, yield 0.74 g (1.82 mmol, 73%), colorless needles, mp 278–281 °C. ^{1}H NMR (400 MHz, CD_3CN): δ = 0.99 (t, *J* = 7 Hz, 6H, CH_3), 3.22–3.28 (m, 4H, NC$\mathbf{H_2}$$CH_3$), 4.33 (s, 4H,

C=CC**H₂**N), 5.23 (s, 2H, C**H**), 6.99, 7.35 (AA'BB'-system, 8H, CH_{ar}). ^{13}C NMR (100 MHz, CD_3CN): δ = 8.8 (CH_3), 49.3 (CH_2), 67.8 (CH_2), 69.2 (CH), 124.5 (CH_{ar}), 125.8 (CH_{ar}), 146.1 (C_q), 146.8 (C_q). MS (ESI^+): *m/z* (%) = 302 (100). Anal. Calcd for $C_{22}H_{24}ClNO_4$ (401.9): C, 65.75; H, 6.02; N, 3.49. Found: C, 65.57; H, 6.01; N, 3.39.

***N*,*N*-Di-*n*-propyl-3,4-(9',10'-dihydro-9',10'-anthraceno)-3-pyrrolinium perchlorate** (**2b**): Prepared from dibenzobarrelene **1** (1.17 g, 3.00 mmol) according to **GP-1**, yield 1.06 g (2.45 mmol, 82%). White crystalline solid, mp 248–249 °C. ^1H NMR (400 MHz, $(CD_3)_2CO$): δ = 0.82 (t, J = 7 Hz, 6H, CH_3), 1.51–1.57 (m, 4H, C$\mathbf{H_2}$$CH_3$), 2.46–2.50 (m, 4H, NC$\mathbf{H_2}$$CH_2$), 4.73 (s, 4H, C=CC$\mathbf{H_2}$N), 5.38 (s, 2H, C**H**), 6.99, 7.38 (AA'BB'-system, 8H, CH_{ar}). ^{13}C NMR (100 MHz, $(CD_3)_2CO$): δ = 11.3 (CH_3), 17.8 (CH_2), 49.8 (CH_2), 66.9 (CH_2), 69.2 (CH), 124.8 (CH_{ar}), 126.1 (CH_{ar}), 146.7 (C_q), 147.2 (C_q). MS (ESI^+): *m/z* (%) = 330 (100). Anal. Calcd for $C_{24}H_{28}ClNO_4$ (429.9): C, 67.05; H, 6.56; N, 3.26. Found: C, 67.22; H, 6.88; N, 3.04.

***N*,*N*-Di-*n*-propyl-3,4-(9',10'-dihydro-9',10'-anthraceno)-3-pyrrolinium chloride** (**2b•Cl**): Obtained quantitatively (0.55 g, 1.50 mmol) by ion exchange of the perchlorate **2b** (0.64 g, 1.50 mmol; eluent: acetonitrile; DOWEX resin). White solid, mp 211–212 °C. ^1H NMR (400 MHz, CD_3OD): δ = 0.78 (t, J = 7 Hz, 6H, CH_3), 1.16–1.34 (m, 8H, C$\mathbf{H_2}$C$\mathbf{H_2}$$CH_3$), 3.28–3.30 (m, 4H, NC$\mathbf{H_2}$$CH_2$, overlapped with solvent signal), 4.48 (s, 4H, CC$\mathbf{H_2}$N), 5.12 (s, 2H, C**H**), 6.99, 7.35 (AA'BB'-system, 8H, CH_{ar}). ^{13}C NMR (100 MHz, CD_3OD): δ = 13.9 (CH_3), 26.5 (CH_2), 50.0 (CH_2), 65.7 (CH_2), 68.7 (CH), 124.6 (CH_{ar}), 126.2 (CH_{ar}), 147.0 (C_q), 147.2 (C_q).

***N*,*N*-Di-isopropyl-3,4-(9',10'-dihydro-9',10'-anthraceno)-3-pyrrolinium perchlorate** (**2c**): Prepared from dibenzobarrelene **1** (1.17 g, 3.00 mmol) according to **GP-1**, the product was further purified by recrystallization from acetone/diethylether to give **2c** (1.11 g, 2.55 mmol, 85%) as white crystalline solid, mp 230–232 °C. ^1H NMR (400 MHz, CD_3CN): δ = 1.02 (d, J = 6 Hz, 12H, CH_3), 3.63–

3.72 [m, 2H, NC**H**($(CH_3)_2$], 4.28 (s, 4H, C=CC**H₂**N), 5.21 (s, 2H, C**H**), 6.98, 7.35 (AA'BB'-system, 8H, CH_{ar}). ^{13}C NMR (100 MHz, CD_3CN): δ = 16.2 (CH_3), 49.4 (CH), 61.7 (CH_2), 64.1 (CH), 124.5 (CH_{ar}), 125.8 (CH_{ar}), 146.7 (C_q), 147.3 (C_q). UV (MeCN): λ_{max} (log ε) = 272 (3.86), 280 (4.02). MS (ESI$^+$): *m/z* (%) = 330 (100). Anal. Calcd for $C_{24}H_{28}ClNO_4$ (429.9): C, 67.05; H, 6.56; N, 3.26. Found: C, 66.62; H, 6.51; N, 3.33.

***N*,*N*-Di-*n*-butyl-3,4-(9',10'-dihydro-9',10'-anthraceno)-3-pyrrolinium perchlorate** (**2d**): Prepared from dibenzobarrelene **1** (1.60 g, 4.08 mmol) according to **GP-1**, yield 1.46 g (3.18 mmol, 78%), colorless prisms, mp 173–174 °C. ^{1}H NMR (400 MHz, $(CD_3)_2CO$): δ = 0.75 (t, *J* = 7 Hz, 6H, CH_3), 1.20–1.26 (m, 4H, C**H_2**CH_3), 1.42–1.46 (m, 4H, C**H_2**CH_2CH_3), 3.52–3.56 (m, 4H, NC**H_2**CH_2), 4.72 (s, 4H, C=CC**H_2**N), 5.39 (s, 2H, C**H**), 6.99, 7.39 (AA'BB'-system, 8H, CH_{ar}). ^{13}C NMR (100 MHz, $(CD_3)_2CO$): δ = 14.1 (CH_3), 20.7 (CH_2), 26.5 (CH_2), 49.8 (CH_2), 65.6 (CH_2), 69.0 (CH), 124.8 (CH_{ar}), 126.1 (CH_{ar}), 144.6 (C_q), 146.9 (C_q). UV (CH_2Cl_2): λ_{max} (log ε) = 273 (4.00), 280 (4.17). MS (ESI$^+$): *m/z* (%) = 358 (100). Anal. Calcd for $C_{26}H_{32}ClNO_4$ (458.0): C, 68.18; H, 7.04; N, 3.06. Found: C, 68.22; H, 7.12; N, 3.05.

***N*,*N*-Di-*n*-butyl-3,4-(9',10'-dihydro-9',10'-anthraceno)-3-pyrrolinium chloride** (**2d-Cl**): Obtained quantitatively (1.37 g, 3.47 mmol) as a white powder by ion exchange of the perchlorate **2d** (1.59 g, 3.47 mmol; eluent: acetonitrile; DOWEX resin). Mp 194–195 °C. ^{1}H NMR (400 MHz, CD_3CN): δ = 0.78 (t, *J* = 7 Hz, 6H, CH_3), 1.16–1.34 (m, 8H, C**H_2**C**H_2**CH_3), 3.28–3.30 (m, 4H, NC**H_2**CH_2, overlapped with the solvent signal), 4.48 (s, 4H, C=CC**H_2**N), 5.12 (s, 2H, C**H**), 6.99, 7.35 (AA'BB'-system, 8H, CH_{ar}). ^{13}C NMR (100 MHz, CD_3CN): δ = 13.9 (CH_3), 20.8 (CH_2), 26.5 (CH_2), 50.0 (CH_2), 65.7 (CH_2), 68.7 (CH), 126.2 (CH_{ar}), 126.7 (CH_{ar}), 144.6 (C_q), 147.0 (C_q).

***N*-4-Benzyl-*N*-methyl-3,4-(9',10'-dihydro-9',10'-anthraceno)-3-pyrrolinium perchlorate** (**2e**): Prepared from dibenzobarrelene **1** (0.30 g, 0.75 mmol) according

to **GP-1**, the precipitated solid was further purified by column chromatography (SiO_2; dichloromethane/methanol 20:1, v/v; R_f = 0.3). Yield 0.17 g (0.37 mmol, 49%), colorless prisms, mp 163–166 °C. ^{1}H NMR (400 MHz, $(CD_3)_2CO$): δ = 3.21 (s, 3H, C**H₃**), 4.59 (d, *J* = 12 Hz, 2H, C=CC**H**HN), 5.04 (d, *J* = 12 Hz, 2H, C=CCH**H**N), 4.87 (s, 2H, NC**H₂**Ph), 5.36 (s, 2H, C**H**), 6.96–7.00 (m, 4H, CH_{ar}), 7.33–7.36 (m, 4H, CH_{ar}), 7.39–7.45 (m, 3H, CH_{ar}), 7.60–7.62 (m, 2H, CH_{ar}). ^{13}C NMR [100 MHz, $(CD_3)_2CO$]: δ = 49.6 (CH_3), 51.8 (CH_2), 68.5 (CH_2), 70.6 (CH), 124.5 (CH_{ar}), 125.7 (CH_{ar}), 125.7 (CH_{ar}), 129.4 (CH_{ar}), 130.0 (C_q), 131.4 (CH_{ar}), 131.5 (CH_{ar}), 133.0 (CH_{ar}), 146.5 (C_q), 146.7 (C_q). MS (ESI^+): *m/z* (%) = 350 (100). Anal. Calcd for $C_{26}H_{24}ClNO_4$ (449.9): C, 69.41; H, 5.38; N, 3.11. Found: C, 69.09; H, 5.54; N, 3.15.

***N*-Cyclohexyl-*N*-methyl-3,4-(9',10'-dihydro-9',10'-anthraceno)-3-pyrrolinium perchlorate** (**2f**): Prepared from dibenzobarrelene **1** (0.40 g, 1.01 mmol) according to **GP-1**, yield 0.21 g (0.47 mmol, 47%), colorless crystalline solid, mp 196–198 °C. ^{1}H NMR (400 MHz, $(CD_3)_2CO$): δ = 1.34–2.13 (m, 10H, cyclohexyl-CH_2), 3.03 (s, 3H, C**H₃**), 3.69 (tt, *J* = 12, 3 Hz, 1H, NC**H**), 4.62 (d, *J* = 12 Hz, 2H, NC**H**H), 4.83 (d, *J* = 12 Hz, 2H, NCH**H**), 5.38 (s, 2H, C**H**), 6.97–7.00 (m, 4H, CH_{ar}), 7.36–7.39 (m, 4H, CH_{ar}). ^{13}C NMR [100 MHz, $(CD_3)_2CO$]: δ = 25.4 (CH_3), 25.8 (CH_2), 27.8 (CH_2), 47.4 (CH_2), 49.6 (CH), 70.3 (CH_2), 74.9 (CH), 124.5 (CH_{ar}), 125.7 (CH_{ar}), 146.6 (C_q), 146.9 (C_q). MS (ESI^+): *m/z* (%) = 342 (100). Anal. Calcd for $C_{26}H_{24}ClNO_4$ (442.0): C, 67.94; H, 6.39; N, 3.17. Found: C, 68.04; H, 6.55; N, 3.15.

***N*-(4''-Benzoylphenyl)-*N*-methyl-3,4-(9',10'-dihydro-9',10'-anthraceno)-3-pyrrolinium perchlorate** (**2g**): Prepared from dibenzobarrelene **1** (0.59 g, 2.18 mmol) according to **GP-1** using 0.5 equiv of DBU catalyst (0.17 g, 1.09 mmol) as colorless cubes (0.87 g, 1.54 mmol, 68%), mp 156–158 °C. ^{1}H NMR (400 MHz, $(CD_3)_2CO$): δ = 0.71 (t, *J* = 7 Hz, 3H, CH_3), 1.19–1.25 (m, 2H, CH_2), 1.44–1.48 (m, 2H, CH_2), 3.65–3.70 (m, 2H, N**CH₂**CH₂), 4.78 (d, *J* = 12 Hz, 2H, C=CC**H**HN), 4.98 (s,

2H, CH_2), 5.12 (d, *J* = 12 Hz, 2H, C=CCH**H**N), 5.30 (s, 2H, CH_2), 6.82–6.97 (m, 4H, CH_{ar}), 7.24–7.35 (m, 4H, CH_{ar}), 7.57–7.79 (m, 9H, CH_{ar}). ^{13}C NMR (100 MHz, $(CD_3)_2CO$): *δ* = 13.6 ($CH_\mathbf{3}$), 20.2 ($CH_\mathbf{2}$), 26.2 ($CH_\mathbf{2}$), 49.2 ($CH_\mathbf{2}$), 66.3 ($CH_\mathbf{2}$), 67.6 ($CH_\mathbf{2}$), 68.3 (CH), 124.4 (CH_{ar}), 125.7 (CH_{ar}), 129.4 (CH_{ar}), 130.7 (CH_{ar}), 131.0 (CH_{ar}), 132.7 (C_q), 133.7 (CH_{ar}), 138.0 (C_q), 139.4 (CH_{ar}), 146.0 (C_q), 146.2 (C_q), 146.8 (C_q), 196.1 (C=O). MS (ESI^+): *m/z* (%) = 497 (100). Anal. Calcd for $C_{37}H_{34}ClNO_5$ (596.1): C, 72.53; H, 5.75; N, 2.35. Found: C, 72.50; H, 5.51; N, 2.44.

***N*,*N*-Di-*n*-propyl-3,4-(9',10'-dihydro-9',10'-anthraceno)-3-pyrrolinium benzoylphenylmethanesulfonate (2b-4):** A solution of silver 4-benzoylphenylmethanesulfonate (0.10 g, 0.27 mmol) in acetonitrile (10 ml) was added dropwise over a period of 5 min to a solution of **2b** (0.10 g, 0.27 mmol) in MeCN (10 ml). The mixture was stirred for 30 min at room temperature and filtered to remove the $AgCl_2$. The filtrate was concentrated in vacuo until ca. 3 ml of the solvent remained. Diethyl ether (ca. 10 ml) was carefully added until crystalline needles started to form. The crystals were allowed to grow for 14 h at room temperature and collected by filtration to give salt **2b-4** as colorless needles (0.12 g, 0.20 mmol, 73%), mp 162–165 °C. ^{1}H NMR (400 MHz, $(CD_3)_2CO$): δ = 0.78 (t, *J* = 7 Hz, 6H, CH_3), 1.40–1.49 (m, 4H, C**H₂**CH₃), 3.41–3.45 (m, 4H, NC**H₂**CH₂), 3.88 (s, 2H, C**H₂**SO₃), 4.67 (s, 4H, C=CC**H₂**N), 5.35 (s, 2H, CH), 6.96, 7.36 (AA'BB'-system, 8H, CH_{ar}), 7.53–7.56 (m, 3H, CH_{ar}), 7.63–7.68 (m, 3H, CH_{ar}) 7.76–7.78 (m, 2H, CH_{ar}). ^{13}C NMR (100 MHz, $(CD_3)_2CO$): δ = 10.0 (CH_3), 16.4 (CH_2), 48.5 (CH_2), 57.7 (CH_2), 65.5 (CH_2), 67.6 (CH), 123.4 (CH_{ar}), 124.7 (CH_{ar}), 128.2 (CH_{ar}), 129.0 (CH_{ar}), 129.6 (CH_{ar}), 130.4 (CH_{ar}), 132.0 (C_q), 145.4 (C_q), 146.0 (C_q). Anal. Calcd for $C_{38}H_{39}NO_4S$ (605.79): C, 75.34; H, 6.49; N, 2.31; S 5.29. Found: C, 75.66; H, 6.08; N, 2.06; S, 5.23.

Synthesis of dibenzosemibullvalene derivatives

General procedure for the irradiation of solutions (GP-2): Solutions of the substrates (10^{-2} to 10^{-3} mol/l) were placed in a 200 ml Duran flask (acetone) or a quartz test tube (other solvents), and argon gas was bubbled through the solution for at least 20 min. The solution was stirred and irradiated for 4–15 h until the starting material was fully converted, as determined by TLC or ^{1}H NMR spectroscopic analysis. After evaporation of the solvent in vacuo, the photolysate was analyzed by ^{1}H NMR spectroscopy, or, in preparative experiments, isolated by recrystallization or column chromatography to obtain the photoproduct.

Irradiation of solid samples: Approximately 15–25 µmol of the crystalline starting material were irradiated in an argon-flushed sealed quartz test tube and irradiated. Subsequently, the mixture was dissolved in dichloromethane. The solvent was removed and the mixture was analyzed by ^{1}H NMR spectroscopy. Alternatively, the crystalline solids were ground to powders and sandwiched between a pair of quartz slides, which were sealed in an argon-flushed polyethylene bag and irradiated. The photolysate was dissolved in dichloromethane, and after the removal of solvent, the mixture was analyzed by ^{1}H NMR spectroscopy.

Irradiation of suspensions: In a 20 ml quartz tube, a suspension of the starting material (0.05–0.16 mmol) in diethylether (10 ml) was added. The mixture was sonicated for 10 min, flushed with argon for 20 min, and irradiated for 12 h. After evaporation of the solvent in vacuo the product mixture was analyzed by ^{1}H NMR spectroscopy.

***rac-N,N*-Diethyl-3,4-{8c,8e-(4b,8b-dihydrodibenzo[*a,f*]cyclopropa[*cd*]pentaleno)}pyrrolidinium perchlorate** (**3a**): Prepared from **2a** (70.0 mg, 0.17 mmol) in acetone solution according to **GP-2**, obtained as colorless cubes (47.0 mg, 0.12 mmol, 67%), mp 197–198 °C. ^{1}H NMR (400 MHz, CD_3CN): δ = 1.05 (t, *J* = 7 Hz, 3H, CH_3), 1.26 (t, *J* = 7 Hz, 3H, CH_3), 2.88–2.95 (m, 1H, CH_2), 2.97–3.04 (m, 1H, CH_2), 3.32–3.37 (m, 2H, CH_2), 3.53 (d, *J* = 13 Hz, 1H, NC**H**H), 3.70 (d, *J* = 13 Hz, 1H, NC**H**H), 4.01 (s, 1 H, CH), 4.25 (d, *J* = 13 Hz, 1H, NCH**H**), 4.55 (d, *J* = 13 Hz, 1H, NCH**H**), 4.91 (s, 1H, CH), 6.98–7.01 (m, 2H, CH_{ar}), 7.07–7.20 (m, 2H, CH_{ar}), 7.27–7.40 (m, 4H, CH_{ar}). ^{13}C NMR (100 MHz, CD_3CN): δ = 8.5 (CH_3), 8.8 (CH_3), 49.3 (CH_2), 55.1 (CH_2), 57.2 (CH_2), 59.6 (CH_2), 61.5 (C_q), 67.1 (CH), 68.2 (C_q), 79.9 (CH), 122.4 (CH_{ar}), 123.4 (CH_{ar}), 125.8 (CH_{ar}), 125.9 (CH_{ar}), 127.7 (CH_{ar}), 128.3 (CH_{ar}), 128.4 (CH_{ar}), 129.0 (CH_{ar}), 136.9 (C_q), 146.0 (C_q), 146.7 (C_q), 152.0 (C_q).

***rac-N,N*-Di-*n*-butyl-3,4-{8c,8e-(4b,8b-dihydrodibenzo[*a,f*]cyclopropa[*cd*]pentaleno)}pyrrolidinium perchlorate** (**3b**): Prepared from **2b** (120 mg, 0.28 mmol) in the aceton solution according to **GP-2**, obtained as colorless crystals (92 mg, 0.21 mmol, 77%), mp 181–182 °C. ^{1}H NMR (400 MHz, CD_3CN): δ = 0.55 (t, *J* = 7 Hz, 3H, CH_3), 0.94 (t, *J* = 7 Hz, 3H, CH_3), 1.40–1.47 (m, 1H, CH_2), 1.48–1.57 (m, 1H, CH_2), 1.60–1.74 (m, 2H, CH_2), 2.67–2.74 (m, 1H, CH_2), 2.85–2.89 (m, 1H, CH_2), 3.14–3.28 (m, 2H, CH_2), 3.50 (d, *J* = 13 Hz, 1H, NC**H**HC), 3.73 (d, *J* = 13 Hz, 1H, NC**H**HC), 4.03 (s, 1 H, CH), 4.23–4.26 (dd, *J* = 13, 2 Hz, 1H, NCH**H**C), 4.55–4.59 (dd, *J* = 13, 2 Hz, 1H, NCH**H**C), 4.91 (s, 1H, CH), 7.07–7.14 (m, 2H, CH_{ar}), 7.14–7.18 (m, 2H, CH_{ar}), 7.27–7.31 (m, 2H, CH_{ar}), 7.33–7.35 (m, 1H, CH_{ar}), 7.38–7.40 (m, 1H, CH_{ar}). ^{13}C NMR (100 MHz, CD_3CN): δ = 10.6 (CH_3), 10.8 (CH_3), 16.7 (CH_2), 17.9 (CH_2), 55.4 (CH_2), 57.3 (CH_2), 61.5 (CH_2), 61.6 (CH_2), 61.9 (C_q), 67.3 (CH), 69.3 (C_q), 70.2 (CH), 122.5 (CH_{ar}), 123.3 (CH_{ar}), 125.8

(CH$_{ar}$), 125.9 (CH$_{ar}$), 127.7 (CH$_{ar}$), 128.3 (CH$_{ar}$), 128.4 (CH$_{ar}$), 128.9 (CH$_{ar}$), 136.9 (C$_q$), 137.1 (C$_q$), 152.0 (C$_q$), 154.4 (C$_q$). Anal. Calcd for $C_{24}H_{28}ClNO_4$ (429.9): C, 67.05; H, 6.56; N, 3.26. Found: C, 67.04; H, 6.60; N, 3.34.

***rac-N,N*-Di-isopropyl-3,4-{8c,8e-(4b,8b-dihydrodibenzo[*a,f*]cyclopropa[*cd*]pentaleno)}pyrrolidinium perchlorate** (**3c**): Prepared from **2c** (70.0 mg, 0.16 mmol) in acetone solution according to **GP-2**, obtained as colorless crystals (55.7 mg, 0.13 mmol, 80%), mp 127–129 °C. ^{1}H NMR (400 MHz, CD_3CN): δ = 0.99 (t, *J* = 7 Hz, 6H, CH_3), 1.05 (t, *J* = 7 Hz, 3H, CH_3), 1.26 (t, *J* = 7 Hz, 3H, CH_3), 2.88–2.95 (m, 1H, CH), 2.97–3.04 (m, 1H, CH), 3.25 (d, *J* = 13 Hz, 1H, NC**H**H), 3.36 (d, *J* = 13 Hz, 1H, NC**H**H), 4.01 (s, 1 H, CH), 4.25 (d, *J* = 13 Hz, 1H, NCH**H**), 4.55 (d, *J* = 13 Hz, 1H, NCH**H**), 6.98–7.01 (m, 2H, CH$_{ar}$), 7.09–7.13 (m, 1H, CH$_{ar}$), 7.15–7.18 (m, 1H, CH$_{ar}$), 7.27–7.30 (m, 1H, CH$_{ar}$), 7.32–7.39 (m, 3H, CH$_{ar}$). ^{13}C NMR (100 MHz, CD_3CN): δ = 8.5 (CH_3), 8.8 (CH_3), 9.5 (CH_3), 9.6 (CH_3), 49.3 (CH), 54.9 (CH), 55.1 (CH_2), 57.3 (CH_2), 61.6 (C$_q$), 67.1 (CH), 67.8 (C$_q$), 69.9 (CH), 122.4 (CH$_{ar}$), 123.3 (CH$_{ar}$), 125.8 (CH$_{ar}$), 125.9 (CH$_{ar}$), 127.7 (CH$_{ar}$), 128.3 (CH$_{ar}$), 128.4 (CH$_{ar}$), 129.0 (CH$_{ar}$), 136.9 (C$_q$), 146.0 (C$_q$), 146.7 (C$_q$), 154.2 (C$_q$).

***rac-N,N*-Di-*n*-butyl-(*rac*)-4b',8b',8c',8e'-dibenzo[*a,f*]cyclopropa[*cd*]pentaleno-pyrrolidinium perchlorate** (**3d**): Prepared from **2d** (50.0 mg, 0.11 mmol) according to **GP-2** in acetone solution as colorless prisms (42.0 mg, 0.09 mmol, 85%), mp 176–178 °C. ^{1}H NMR (400 MHz, CD_3CN): δ = 0.61 (t, *J* = 7 Hz, 3H, CH_3), 0.74 (t, *J* = 7 Hz, 3H, CH_3), 0.85–0.94 (m, 1H, CH_2), 0.99–1.07 (m, 1H, CH_2), 1.16–1.33 (m, 4H, CH_2), 1.33–1.39 (m, 2H, CH_2), 2.74–2.82 (m, 1H, CH_2), 2.88–2.95 (m, 2H, CH_2), 3.48 (d, *J* = 13 Hz, 1H, NC**H**HC), 3.73 (d, *J* = 13 Hz, 1H, NC**H**HC), 4.04 (s, 1 H, CH), 4.24 (d, *J* = 13 Hz, 1H, NCH**H**C), 4.57 (d, *J* = 13 Hz, 1H, NCH**H**C), 4.91 (s, 1H, CH), 6.97-7.01 (m, 2H, CH$_{ar}$), 7.09–7.17 (m, 4H, CH$_{ar}$), 7.34–7.37 (m, 1H, CH$_{ar}$), 7.39–7.41 (m, 1 H, CH$_{ar}$), 7.38–7.40 (m, 1H, CH$_{ar}$). ^{13}C NMR (100 MHz, CD_3CN): δ = 13.0 (CH_3),

13.6 (CH_3), 20.1 (CH_2), 20.3 (CH_2), 24.8 (CH_2), 25.8 (CH_2), 55.5 (CH_2), 55.7 (CH_2), 59.8 (CH_2), 60.0 (CH_2), 61.9 (C_q), 67.3 (CH), 69.4 (C_q), 70.1 (CH), 122.5 (CH_{ar}), 123.3 (CH_{ar}), 125.8 (CH_{ar}), 125.9 (CH_{ar}), 127.7 (CH_{ar}), 128.3 (CH_{ar}), 128.4 (CH_{ar}), 128.9 (CH_{ar}), 137.0 (C_q), 137.1 (C_q), 152.0 (C_q), 154.5 (C_q). MS (ESI^+): *m/z* (%) = 358 (100). Anal. Calcd for $C_{26}H_{32}ClNO_4$ (458.0): C, 68.18; H, 7.04; N, 3.06. Found: C, 68.22; H, 7.14; N, 3.06.

***rac-N,N*-Di-*n*-butyl-3,4-{8c,8e-(4b,8b-dihydrodibenzo[*a,f*]cyclopropa[*cd*]pentaleno)}pyrrolidinium chloride** (**3d•Cl**): Obtained quantitively from the corresponding perchlorate of **2d** (120 mg, 0.26 mmol) by ion exchange according to **GP-1** as white powder, mp 138–140 °C. ^{1}H NMR (400 MHz, CD_3OD): δ = 0.66 (t, *J* = 7 Hz, 3H, CH_3), 0.99 (t, *J* = 7 Hz, 3H, CH_3), 1.09–1.14 (m, 1H, CH_2), 1.29–1.46 (m, 3H, CH_2), 1.58–1.83 (m, 4H, CH_2), 2.97 (td, *J* = 13, 4 Hz, 1H, CH_2), 3.22 (td, *J* = 13, 4 Hz, 1H, CH_2), 3.83 (td, *J* = 13, 4 Hz, 1H, CH_2), 4.23 (td, *J* = 13, 4 Hz, 1H, CH_2), 4.56–4.78 (m, 4H, NC$\mathbf{H_2}$C), 4.99 (s, 1 H, CH), 5.23 (s, 1H, CH), 7.08–7.18 (m, 4H, CH_{ar}), 7.29–7.38 (m, 3H, CH_{ar}), 7.54–7.56 (m, 1 H, CH_{ar}). ^{13}C NMR (100 MHz, CD_3OD): δ = 13.2 (CH_3), 14.0 (CH_3), 20.1 (CH_2), 20.5 (CH_2), 24.8 (CH_2), 26.5 (CH_2), 55.9 (CH_2), 57.4 (CH_2), 58.1 (CH_2), 58.8 (CH_2), 60.8 (C_q), 67.8 (CH), 68.2 (C_q), 68.4 (CH), 122.3 (CH_{ar}), 123.1 (CH_{ar}), 125.9 (CH_{ar}), 126.0 (CH_{ar}), 127.4 (CH_{ar}), 127.8 (CH_{ar}), 128.1 (CH_{ar}), 128.5 (CH_{ar}), 137.7 (C_q), 138.3 (C_q), 152.3 (C_q), 154.6 (C_q).

***rac-N-Benzyl-N*-methyl-3,4-{8c,8e-(4b,8b-dihydrodibenzo[*a,f*]cyclopropa[*cd*]pentaleno)}pyrrolidinium perchlorate** (**3e^{I}**): Prepared from **2e** (103 mg, 0.23 mmol) in acetone solution according to **GP-2**. The diastereoselectivity between the two isomers **3e^{I}** and **3e^{II}** (57:43) was determined by ^{1}H NMR spectroscopic analysis of the photolysate. The major isomer **3e^{I}** was isolated as white solid (15.0 mg, 0.04 mmol, 15%) by column chromatography (SiO_2;

CH_2Cl_2/MeOH = 20:1, v/v; R_f = 0.30) and crystallized from acetone/Et_2O; mp 134–136 °C. ^{1}H NMR (400 MHz, $(CD_3)_2CO$): δ = 2.90 (s, 3H, CH_3), 4.28 (d, *J* = 13 Hz, 1H, NC**H**H), 4.35 (s, 1H, CH), 4.39 (d, *J* = 13 Hz, 1H, NC**H**H), 4.46 (d, *J* = 13 Hz, 1H, NCH**H**), 4.67 (d, *J* = 13 Hz, 1H, NCH**H**), 4.92 (s, 2H, CH_2), 5.01 (s, 1H, CH), 7.06–7.16 (m, 4H, CH_{ar}), 7.31 (d, *J* = 5 Hz, 1H, CH_{ar}), 7.34 (d, *J* = 5 Hz, 1H, CH_{ar}), 7.43 (d, *J* = 5 Hz, 1H, CH_{ar}), 7.51–7.55 (m, 3H, CH_{ar}), 7.69–7.70 (m, 2H, CH_{ar}). MS (ESI^+): *m/z* (%) = 350 (100). The configuration was confirmed by NOE correlations between the methyl group (δ = 2.90) and the 8b-H (δ = 4.35).

***rac-N-Cyclohexyl-N*-methyl-3,4-{8c,8e-(4b,8b-dihydrodibenzo[*a,f*]cyclopropa[*cd*]pentaleno)}pyrrolidinium perchlorate** (**3f^{I}**): Prepared from **2f** (97.0 mg, 0.22 mmol) in acetone solution according to **GP-2**. The diastereoselectivity between the two isomers **3f^{I}** and **3f^{II}** (62:38) was determined by ^{1}H NMR spectroscopic analysis of the photolysate. The major isomer **3f^{I}** was islated as white needles (17 mg, 0.04 mmol, 18%) by column chromatography (SiO_2; CH_2Cl_2/MeOH = 20:1, v/v; R_f = 0.45) and recrystallization from acetone/hexane, mp 117–118 °C. ^{1}H NMR (400 MHz, $(CD_3)_2CO$): δ = 1.22–2.28 (m, 10H, cyclohexyl-CH_2), 2.89 (s, 3H, CH_3), 3.82 (tt, *J* = 12, 3 Hz, 1H, NCH), 4.04 (d, *J* = 13 Hz, 1H, NC**H**H), 4.18 (d, *J* = 13 Hz, 1H, NC**H**H), 4.20 (s, 1H, CH), 4.66 (d, *J* = 13 Hz, 1H, NCH**H**), 4.91 (d, *J* = 13 Hz, 1H, NCH**H**), 5.02 (s, 1H, CH), 7.06–7.20 (m, 4H, CH_{ar}), 7.33–7.39 (m, 3H, CH_{ar}), 7.46 (d, *J* = 8 Hz, 1H, CH_{ar}). ^{13}C NMR (400 MHz, $(CD_3)_2CO$): δ = 25.6 (CH_2), 25.8 (CH_2), 25.9 (CH_2), 28.5 (CH_2), 28.6 (CH_2), 45.0 (C_q), 57.5 (CH), 61.7 (CH), 67.1 (C_q), 70.7 (CH), 72.7 (CH), 74.0 (CH), 122.4 (CH_{ar}), 123.3 (CH_{ar}), 125.8 (CH_{ar}), 126.1 (CH_{ar}), 127.6 (CH_{ar}), 128.1 (CH_{ar}), 128.3 (CH_{ar}), 128.8 (CH_{ar}), 137.2 (C_q), 137.6 (C_q), 152.1 (C_q), 154.3 (C_q). MS (ESI^+): *m/z* (%) = 342 (100). The configuration was confirmed based on the NOE correlations between the methyl group (δ = 2.89) and the *8b*-H (δ = 4.20).

Phase transfer catalyzed alkylation reactions

a) **Analysis with gas chromatography**: A biphasic mixture of the quaternary ammonium catalyst **3d** or TBAC (0.05 mmol), phenylacetonitrile (**5**, 506 mg, 4.32 mmol) and bromoethane (808 mg, 4.75 mmol) in toluene (0.85 ml) and aqueous KOH solution (4 ml, 60%) was stirred for 2 h at 20 °C. Water (10 ml) and toluene (10 ml) were added and the mixture was thoroughly shaken. After phase separation, the organic layer was analyzed by gas chromatography.

Stationary Phase: HP-5 PhMe-Silica, crosslinked 5%, 25 m.

Mobile Phase: Argon gas.

Flow Pressure: 30 kPa.

Temperature: Oven 220 °C, Injector 250 °C, Detector 250 °C.

Retention time: $PhCH_2CN$ 9.6 min; $PhCH(CH_2CH_3)CN$ 10.6 min.

Three PTC reactions were performed in parallel with a) no catalyst, b) TBAB as the catalyst, or c) **3d** as the catalyst.

NMR-spectroscopically monitored PTC reactions: A biphasic mixture of the quaternary ammonium catalyst (0.03 mmol), the substrate (0.10 mmol), benzylchloride (1.25–1.50 equiv) and 2,7-dimethylnaphthalene (as internal standard, 25.0 μmol) in toluene (5 ml) and aqueous KOH solution (5 ml, 10%) was stirred for 2 h at 20 °C. At the end of reaction, water (10 ml) and toluene (10 ml) were added and the mixture was thoroughly shaken. The separated organic phase was analyzed by ^1H NMR spectroscopy, and the conversion calculated by comparison with the signal of the methyl protons of 2,7-dimethylnaphthalene (δ = 2.49 ppm, in $CDCl_3$). Three reactions were run in parallel with a) no catalyst, b) TBAC as the catalyst or c) **3d** as the catalyst.

^{1}H NMR and ^{13}C NMR spectra

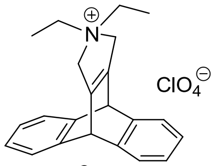

2a

in CD_3CN

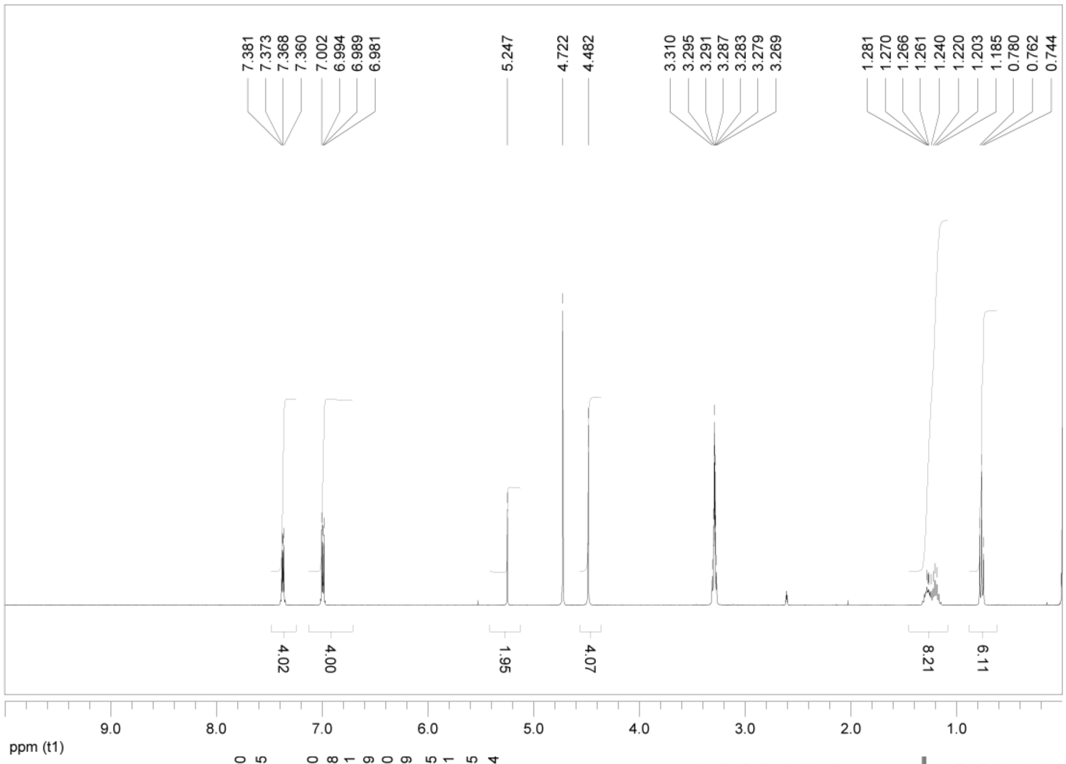

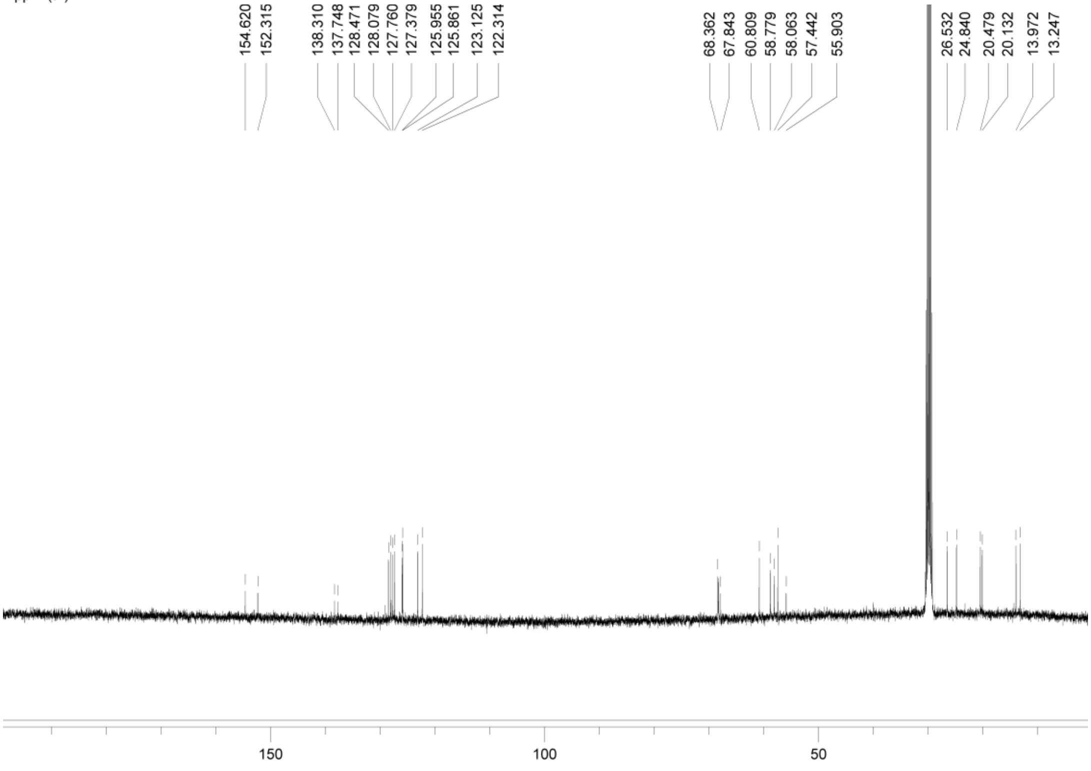

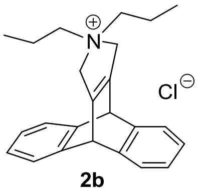

N
Cl

2b (in CD_3OD)

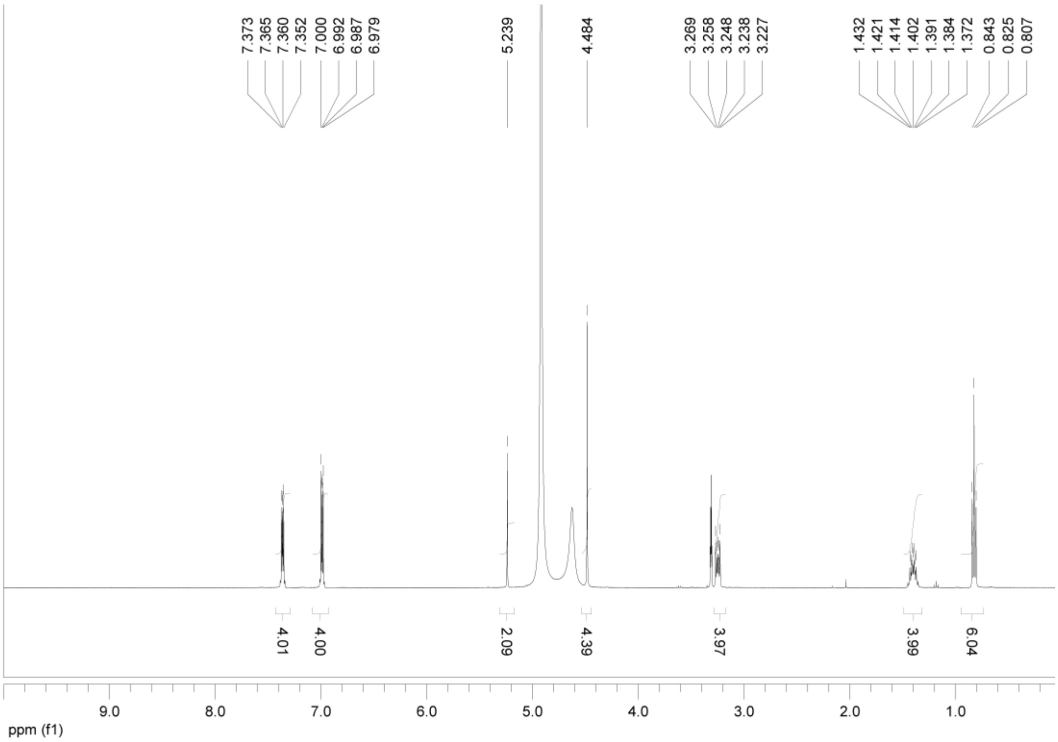

7.373
7.365
7.360
7.352
7.000
6.992
6.987
6.979
5.239
4.484
3.269
3.258
3.248
3.238
3.227
1.432
1.421
1.414
1.402
1.391
1.384
1.372
0.843
0.825
0.807
4.01
4.00
2.09
4.39
3.97
3.99
6.04
9.0
8.0
7.0
6.0
5.0
4.0
3.0
2.0
1.0
ppm (f1)

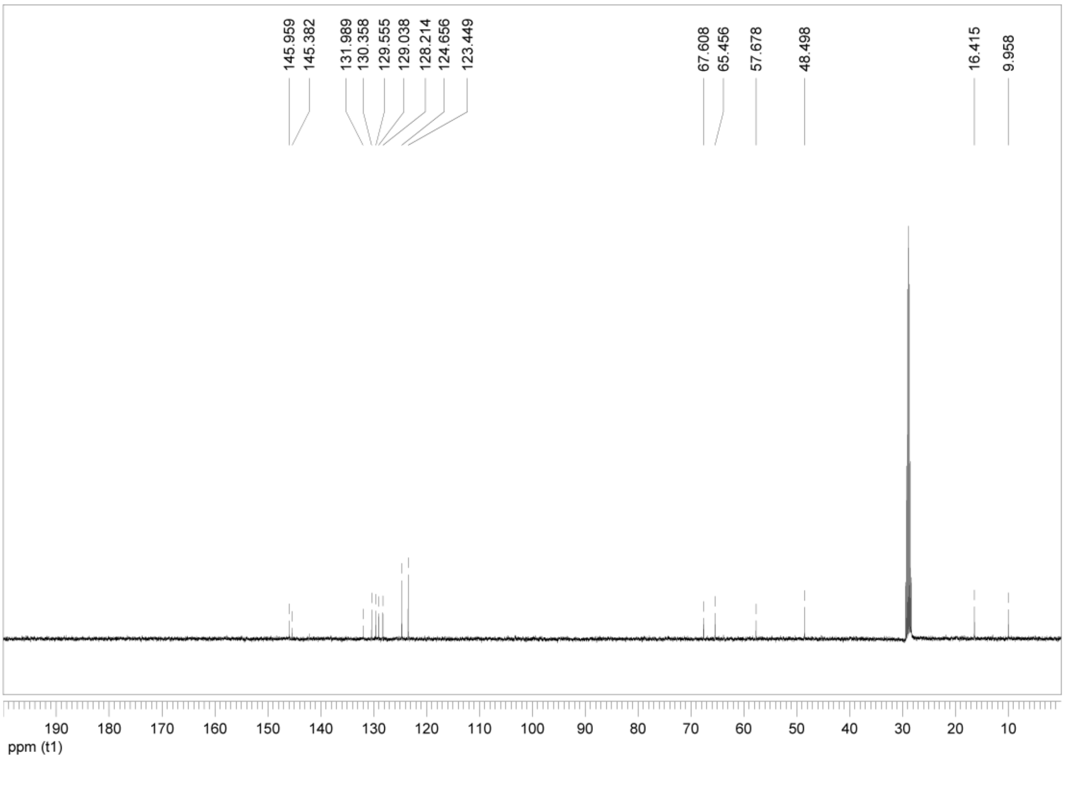

145.959
145.382
131.989
130.358
129.555
129.038
128.214
124.656
123.449
67.608
65.456
57.678
48.498
16.415
9.958
190
180
170
160
150
140
130
120
110
100
90
80
70
60
50
40
30
20
10
ppm (t1)

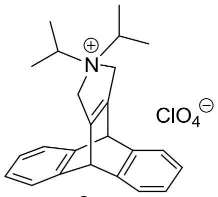
N
ClO4

2c

in $(CD_3)_2CO$

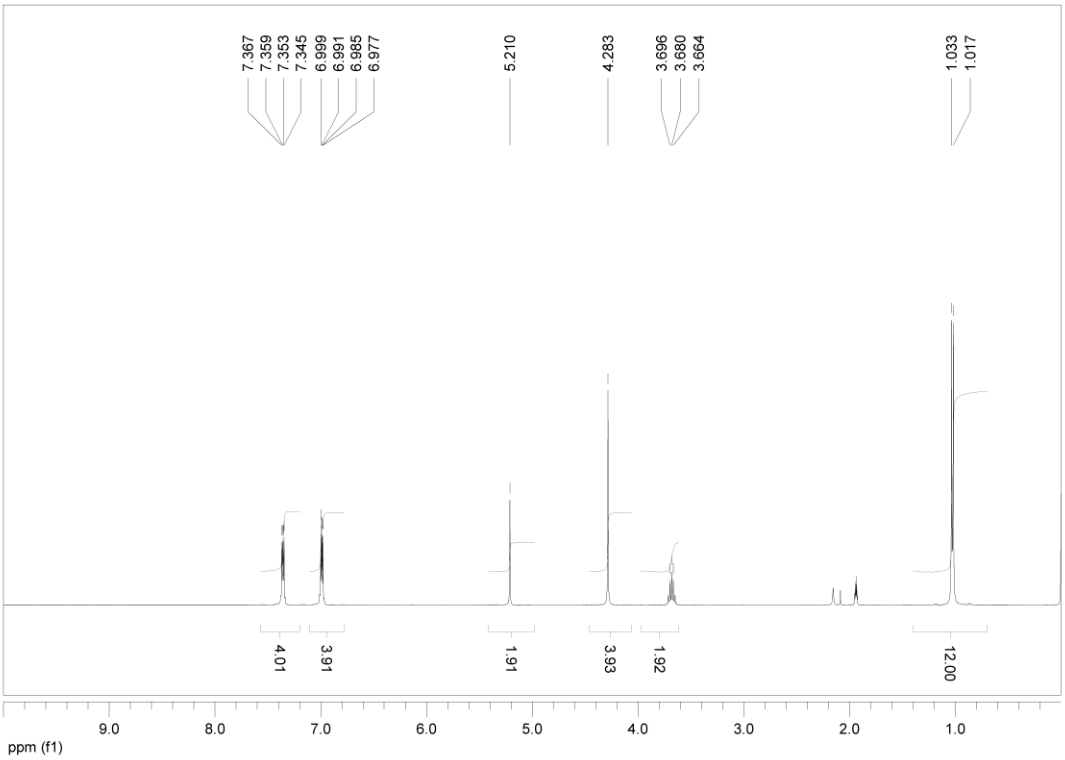
7.367
7.359
7.353
7.345
6.999
6.991
6.985
6.977
5.210
4.283
3.696
3.680
3.664
1.033
1.017
4.01
3.91
1.91
3.93
1.92
12.00
9.0
8.0
7.0
6.0
5.0
4.0
3.0
2.0
1.0
ppm (f1)

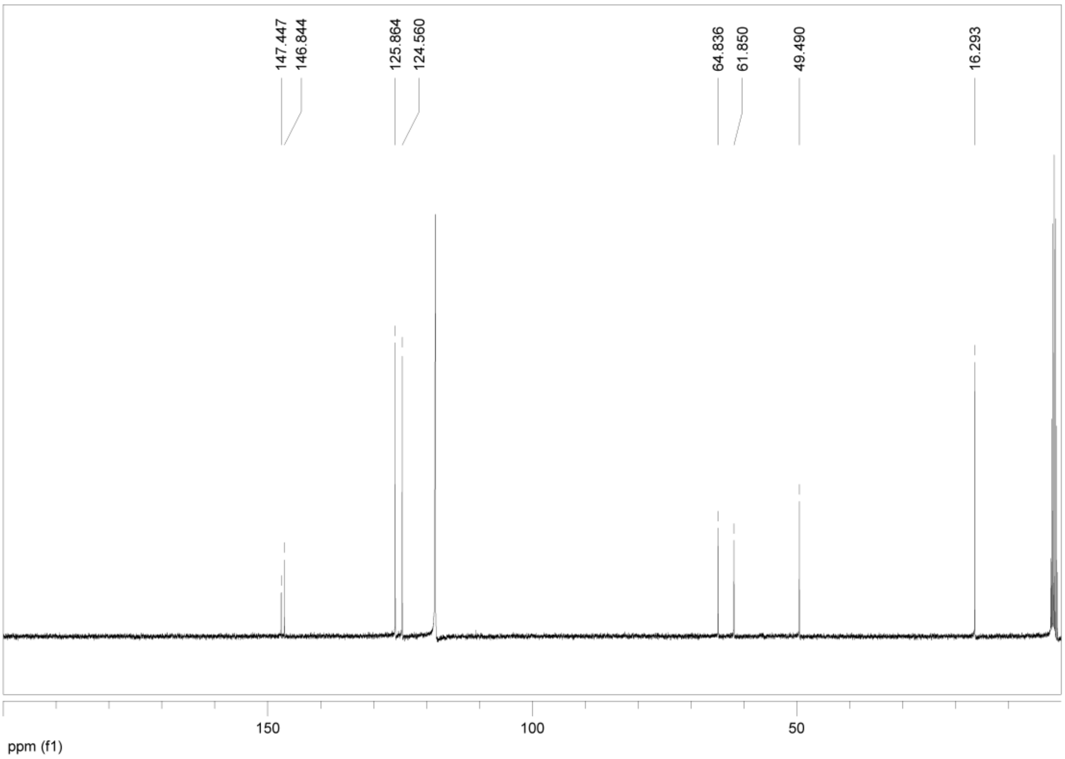
147.447
146.844
125.864
124.560
64.836
61.850
49.490
16.293
150
100
50
ppm (f1)

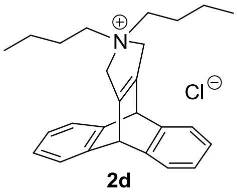

2d in CD_3CN

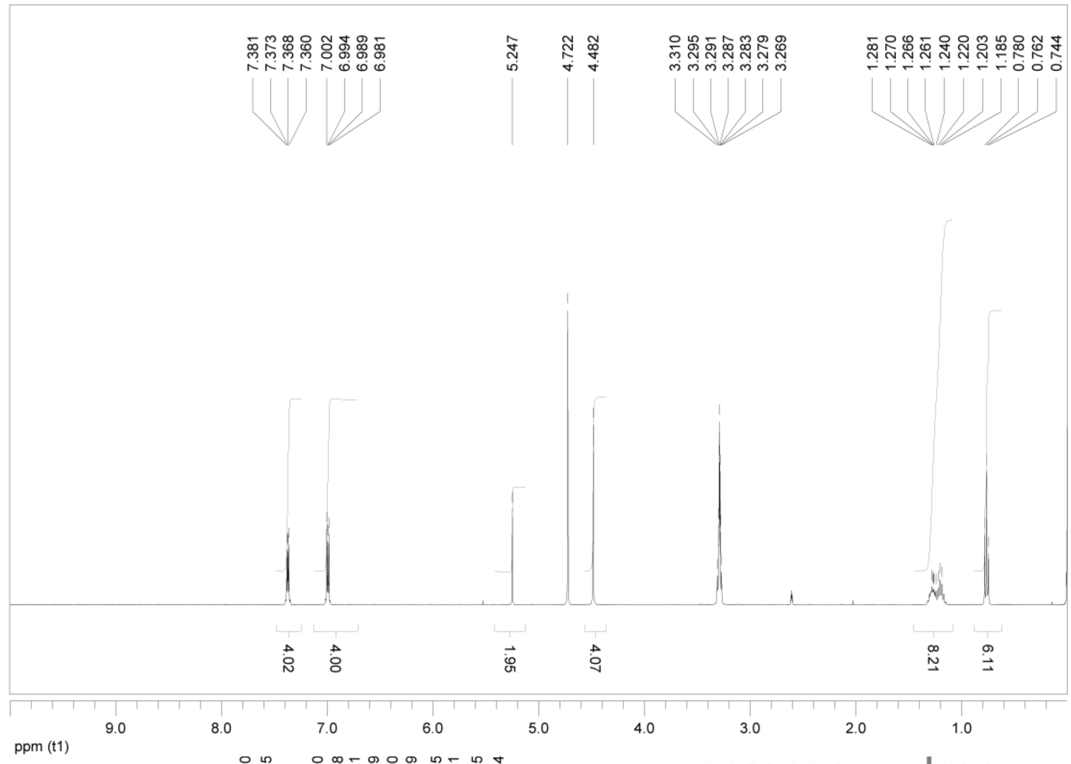
7.381
7.373
7.368
7.360
7.002
6.994
6.989
6.981
5.247
4.722
4.482
3.310
3.295
3.291
3.287
3.283
3.279
3.269
1.281
1.270
1.266
1.261
1.240
1.220
1.203
1.185
0.780
0.762
0.744
4.02
4.00
1.95
4.07
8.21
6.11
9.0
8.0
7.0
6.0
5.0
4.0
3.0
2.0
1.0
ppm (t1)

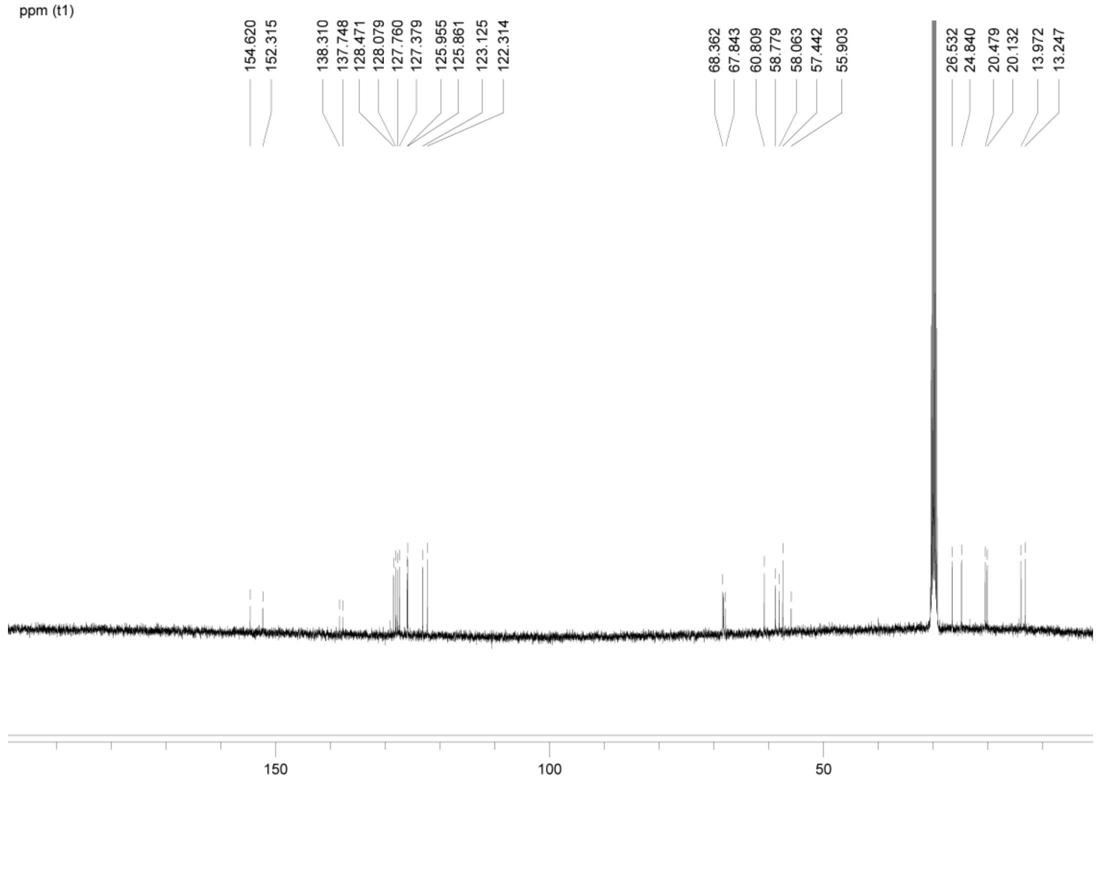
154.620
152.315
138.310
137.748
128.471
128.079
127.760
127.379
125.955
125.861
123.125
122.314
68.362
67.843
60.809
58.779
58.063
57.442
55.903
26.532
24.840
20.479
20.132
13.972
13.247
150
100
50

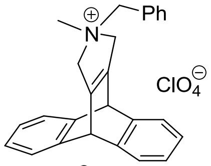
Ph
ClO4

2e

in $(CD_3)_2CO$

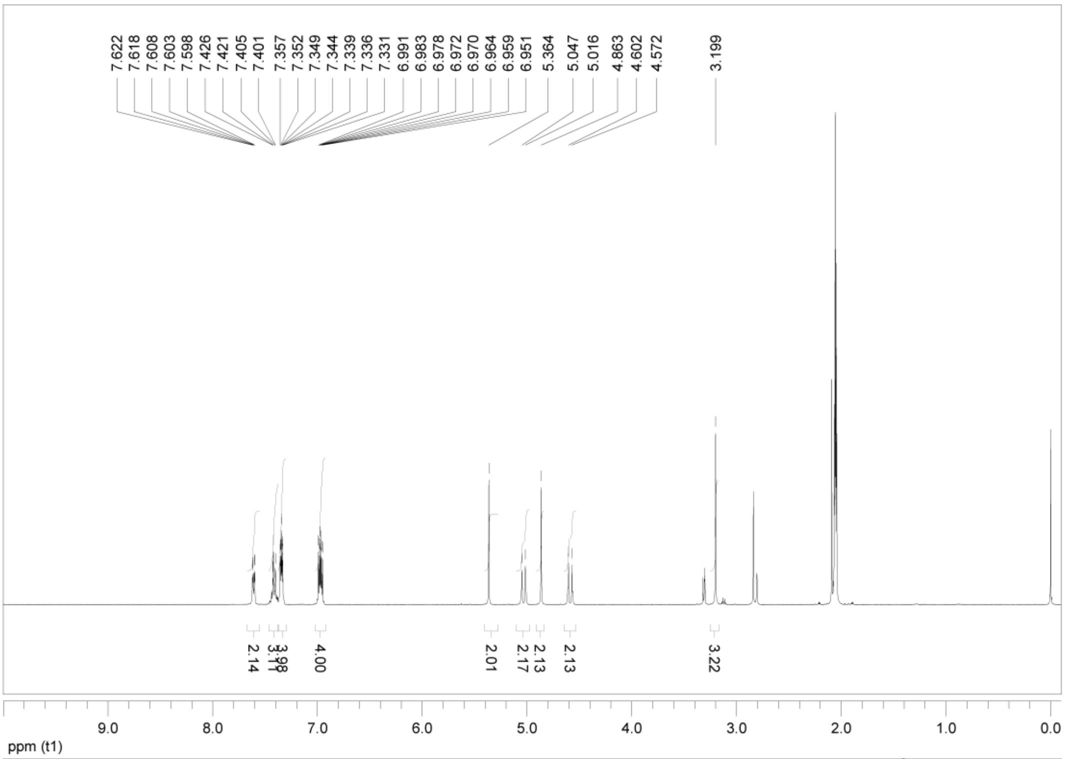
7.622 7.618 7.608 7.603 7.598 7.426 7.421 7.405 7.401 7.357 7.352 7.349 7.344 7.339 7.336 7.331 6.991 6.983 6.978 6.972 6.970 6.964 6.959 6.951 5.364 5.047 5.016 4.863 4.602 4.572 3.199
2.14 3.98 3.11 4.00 2.01 2.17 2.13 2.13 3.22
9.0 8.0 7.0 6.0 5.0 4.0 3.0 2.0 1.0 0.0
ppm (t1)

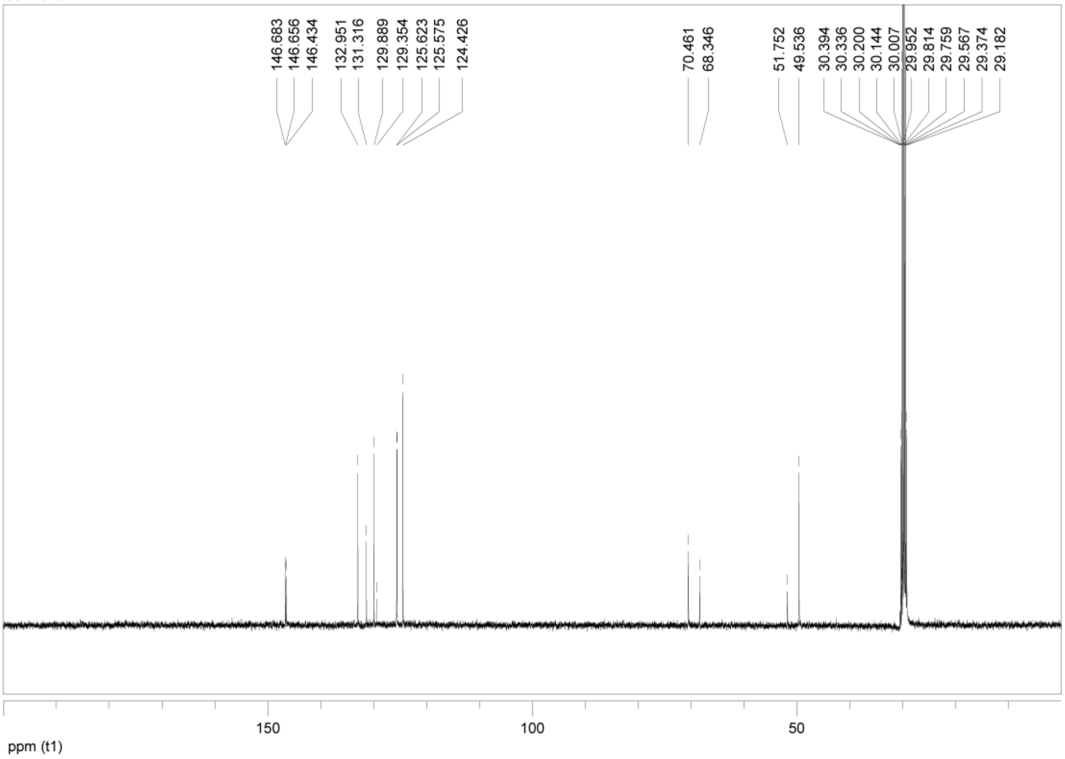
146.683 146.656 146.434 132.951 131.316 129.889 129.354 125.623 125.575 124.426 70.461 68.346 51.752 49.536 30.394 30.336 30.200 30.144 30.007 29.952 29.814 29.759 29.567 29.374 29.182
150 100 50
ppm (t1)

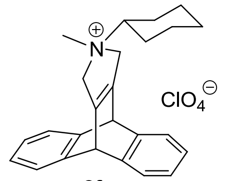

N
ClO4

2f in $(CD_3)_2CO$

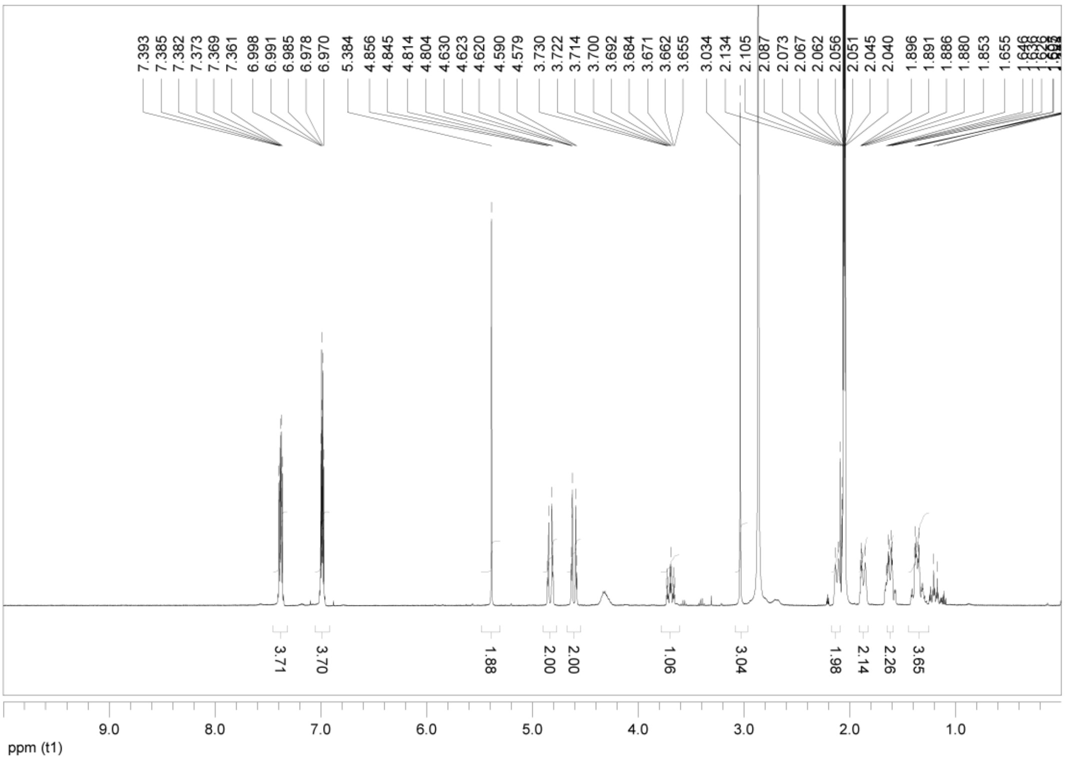

7.393 7.385 7.382 7.373 7.369 7.361 6.998 6.991 6.985 6.978 6.970 5.384 4.856 4.845 4.814 4.804 4.630 4.623 4.620 4.590 4.579 3.730 3.722 3.714 3.700 3.692 3.684 3.671 3.662 3.655 3.034 2.134 2.105 2.087 2.073 2.067 2.062 2.056 2.051 2.045 2.040 1.896 1.891 1.886 1.880 1.853 1.655 1.646
3.71 3.70 1.88 2.00 2.00 1.06 3.04 1.98 2.14 2.26 3.65
9.0 8.0 7.0 6.0 5.0 4.0 3.0 2.0 1.0
ppm (t1)

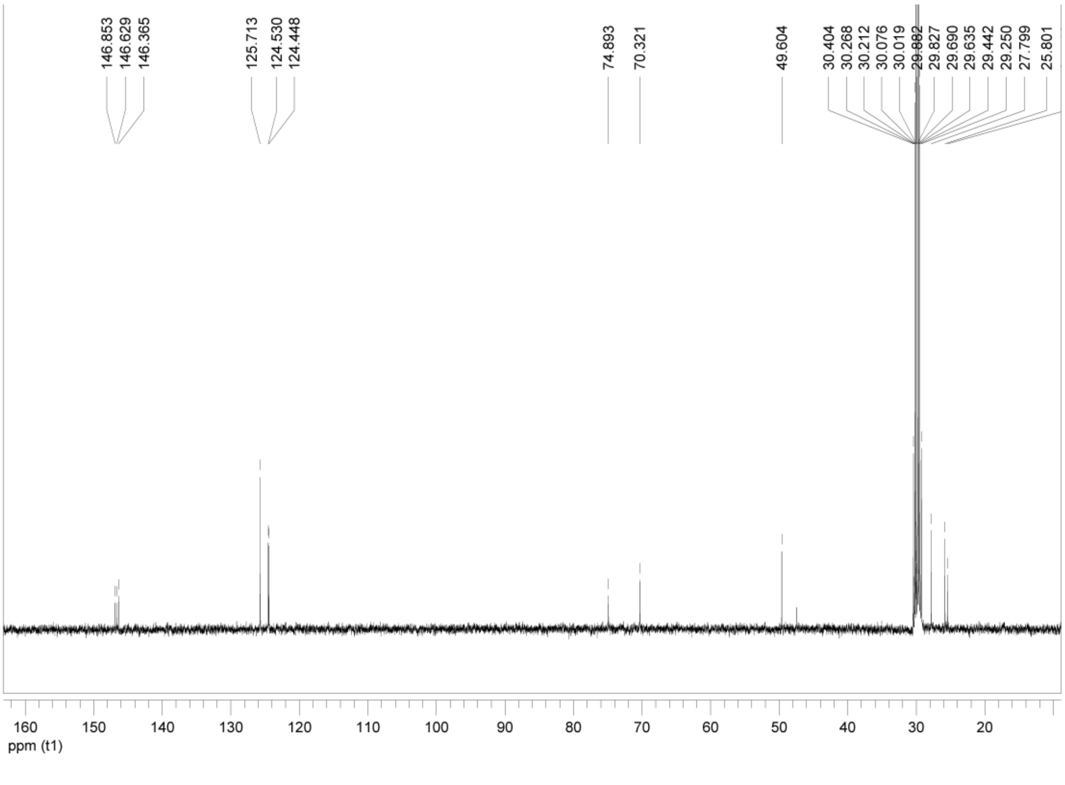

146.853 146.629 146.365 125.713 124.530 124.448 74.893 70.321 49.604 30.404 30.268 30.212 30.076 30.019 29.882 29.827 29.690 29.635 29.442 29.250 27.799 25.801
160 150 140 130 120 110 100 90 80 70 60 50 40 30 20
ppm (t1)

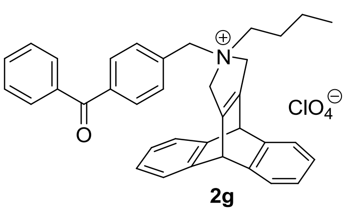

2g

in $(CD_3)_2CO$

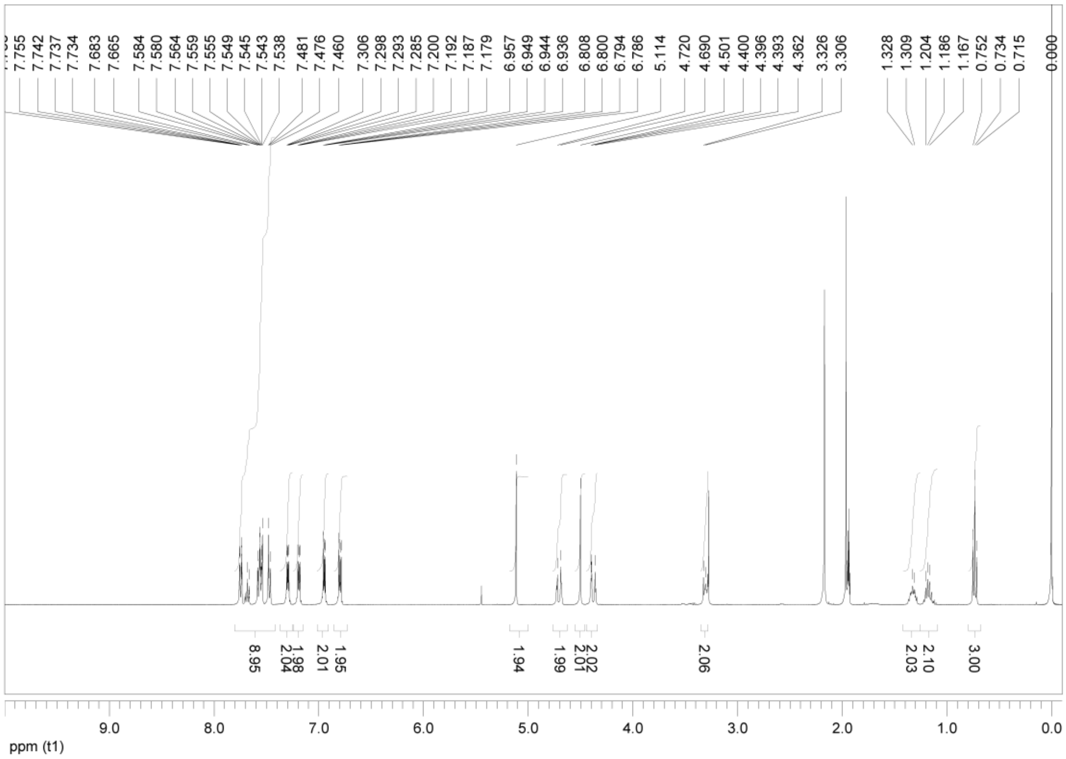

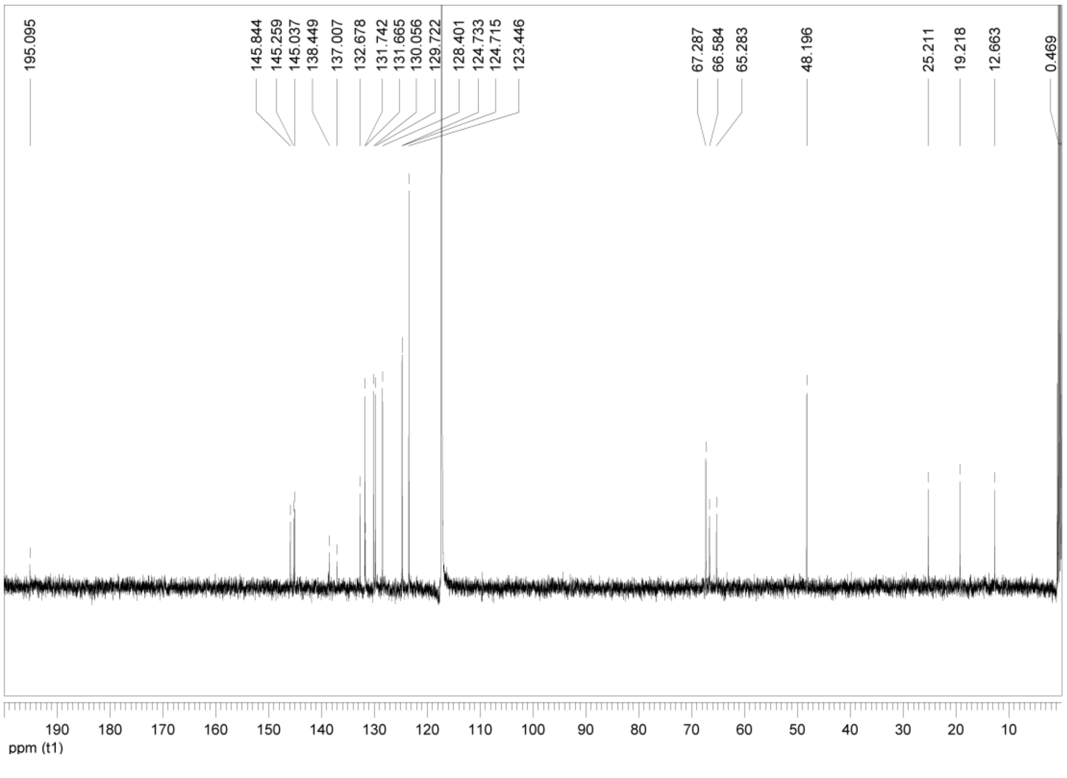

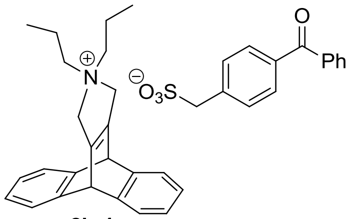

2b-4

in $(CD_3)_2CO$

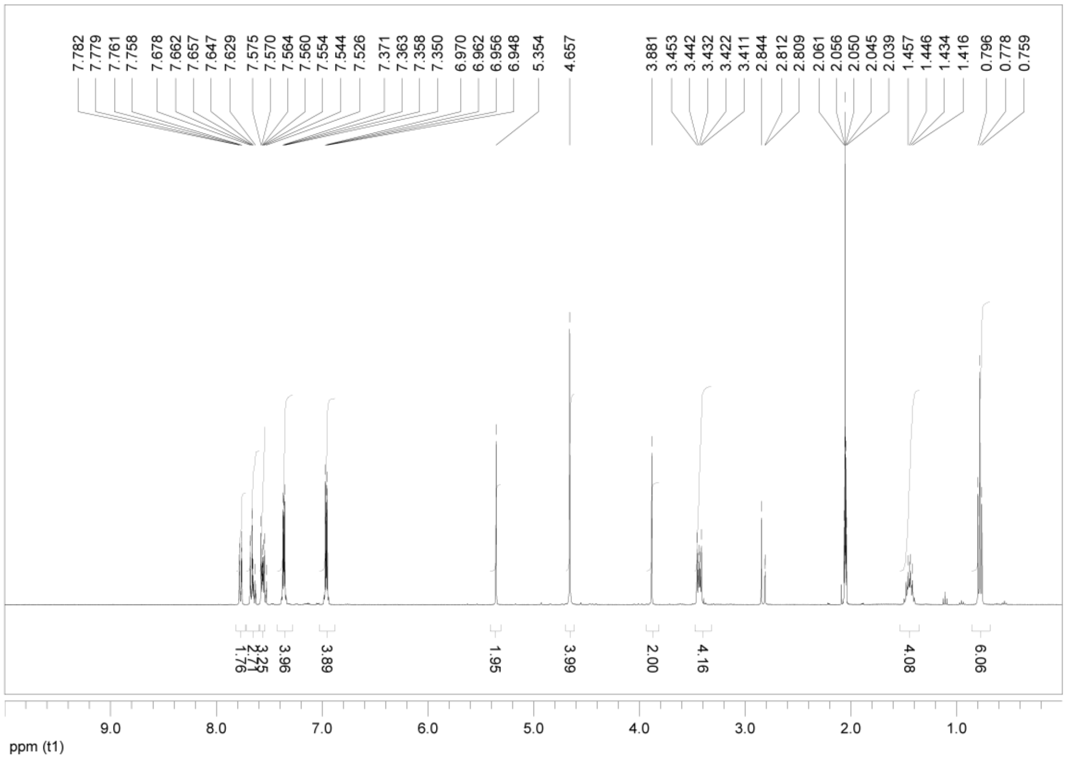

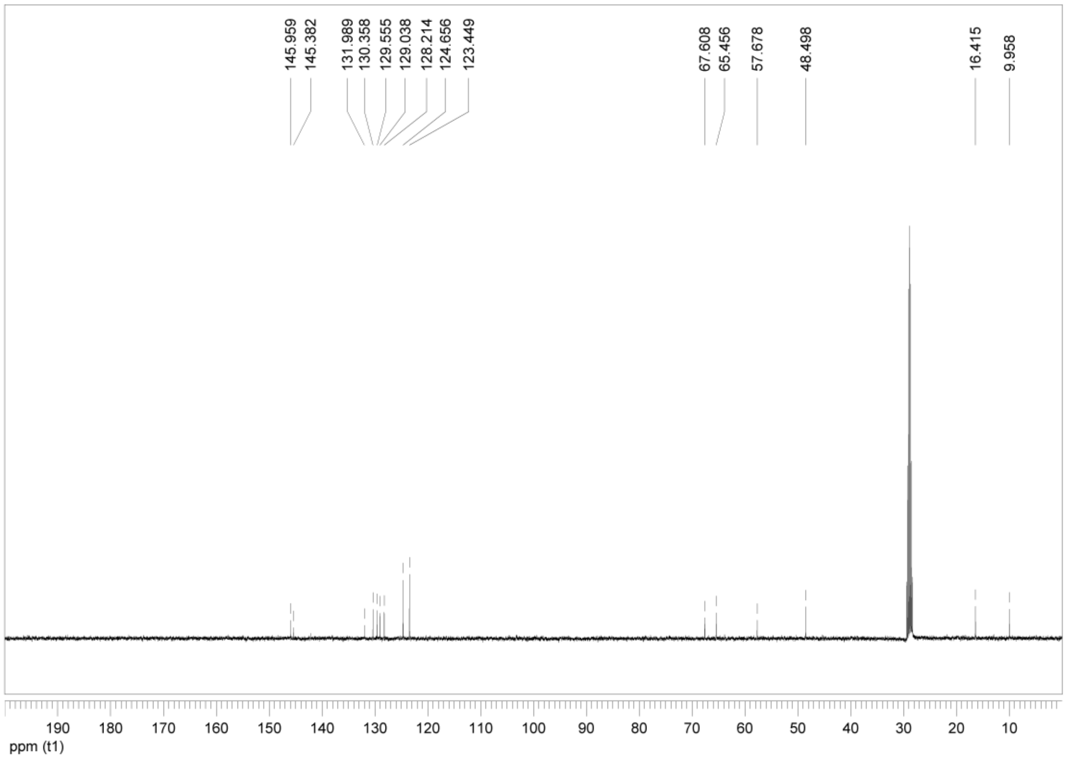

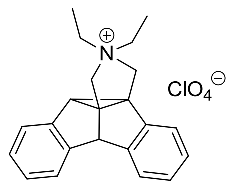

rac-**3a**

in CD_3CN

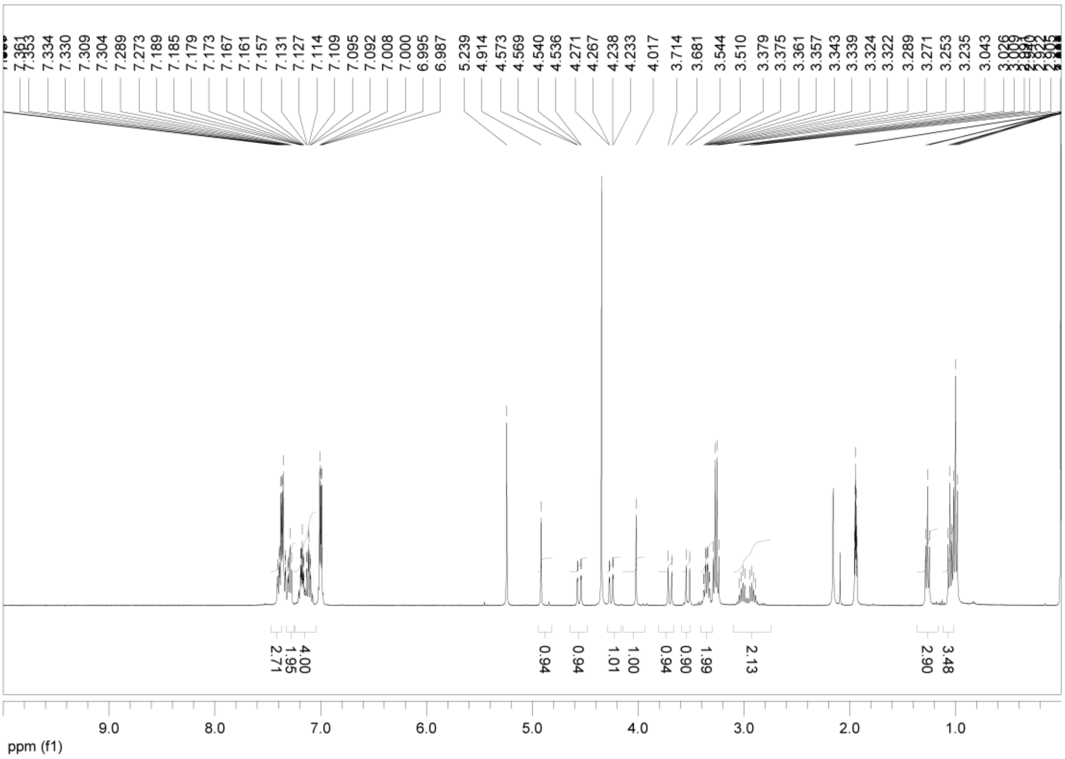

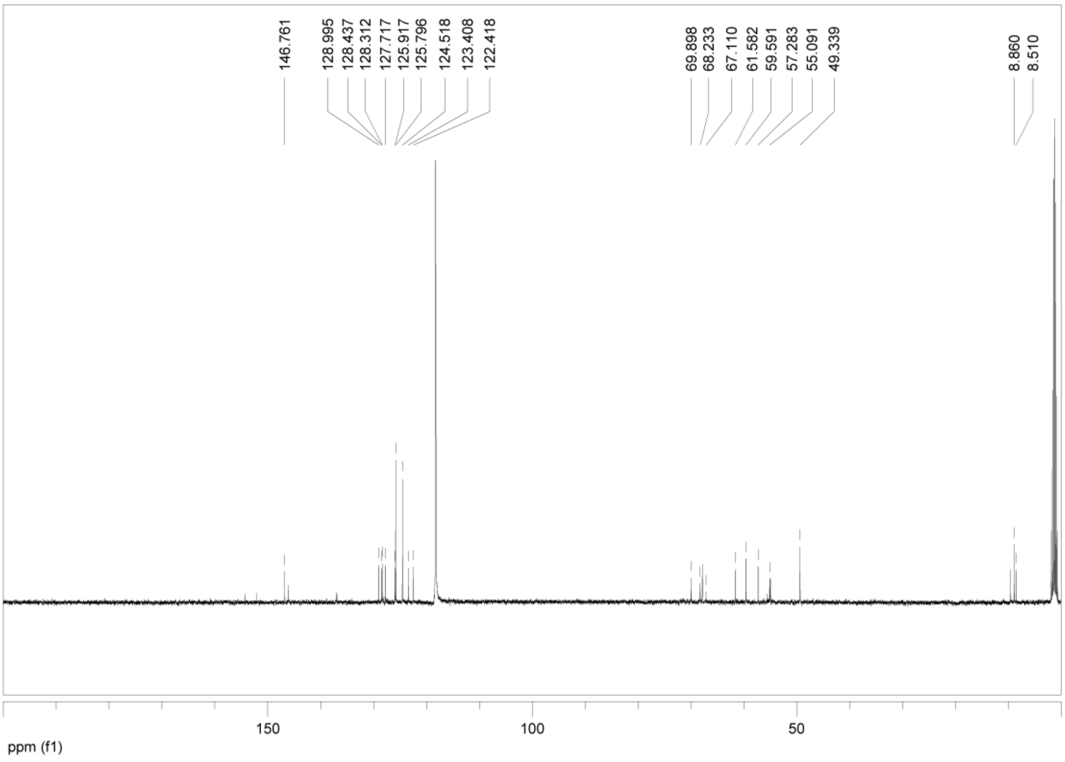

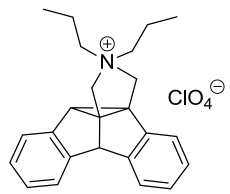

rac-**3b** in CD_3CN

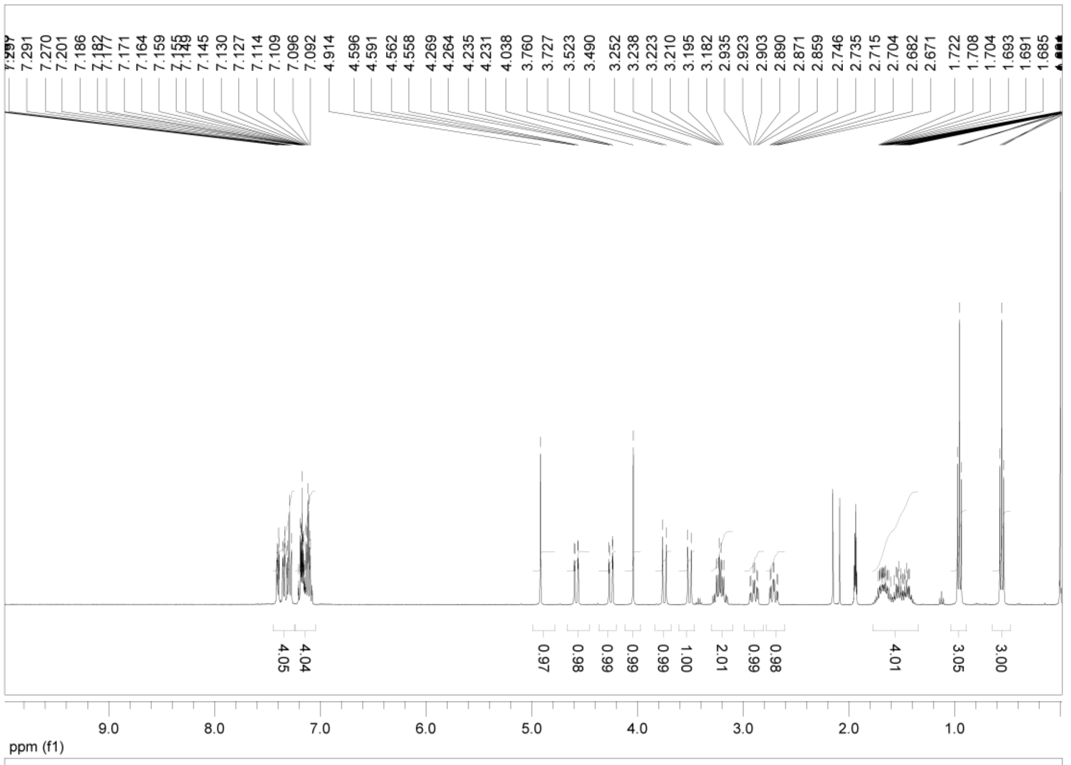

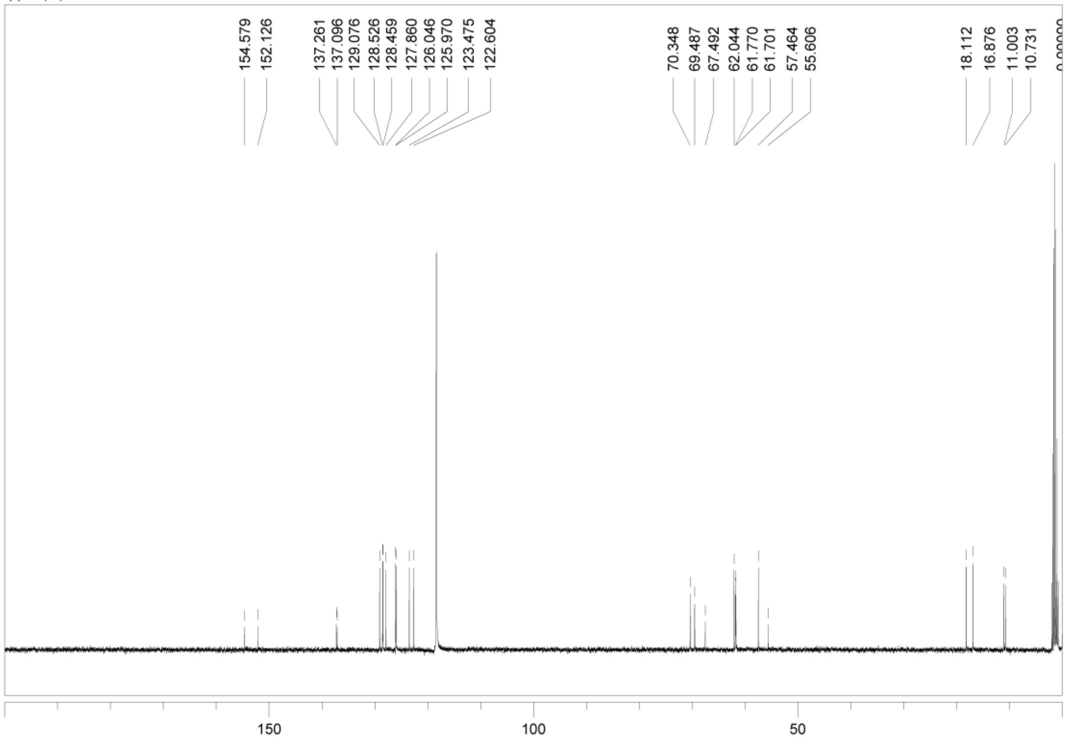

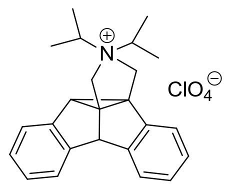

rac-**3c**

in CD_3CN

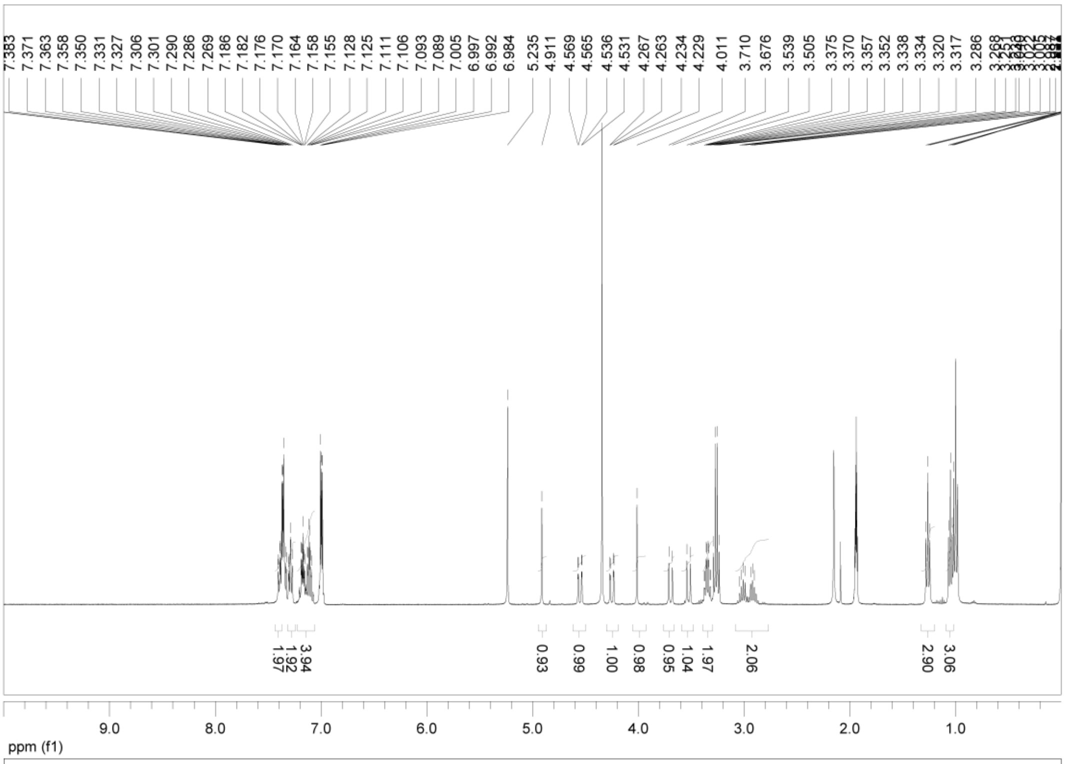

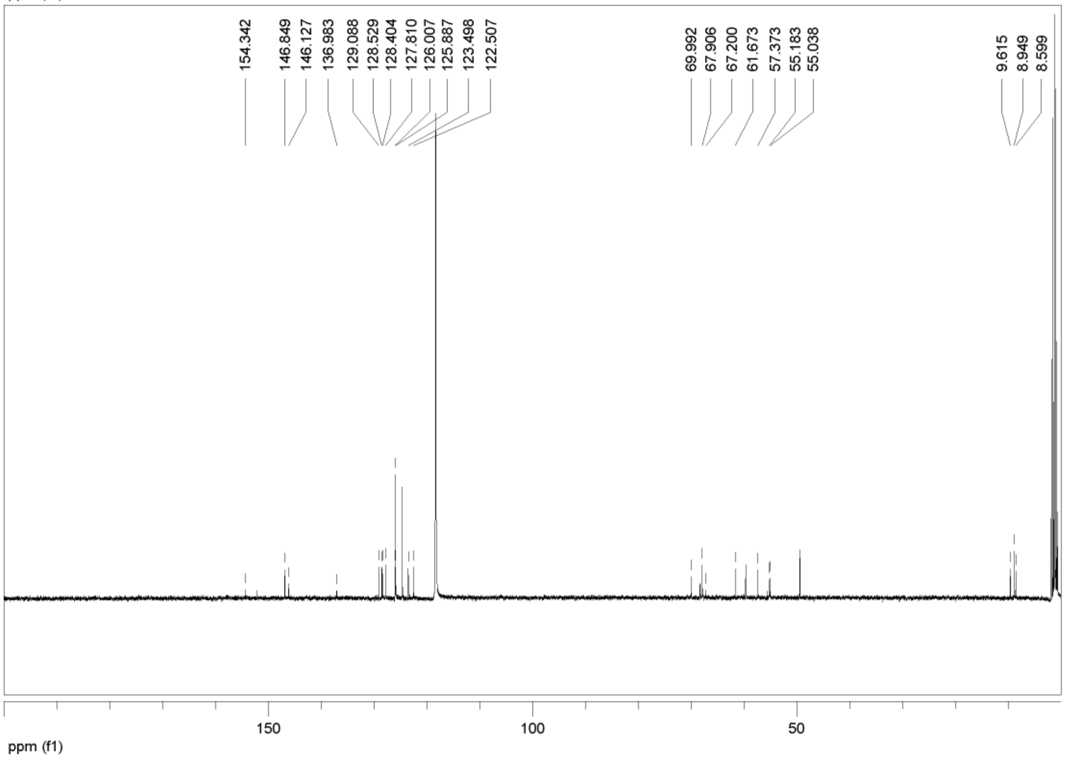

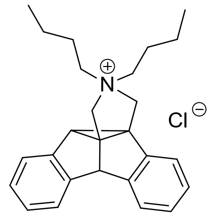

rac-**3d** in CD_3OD

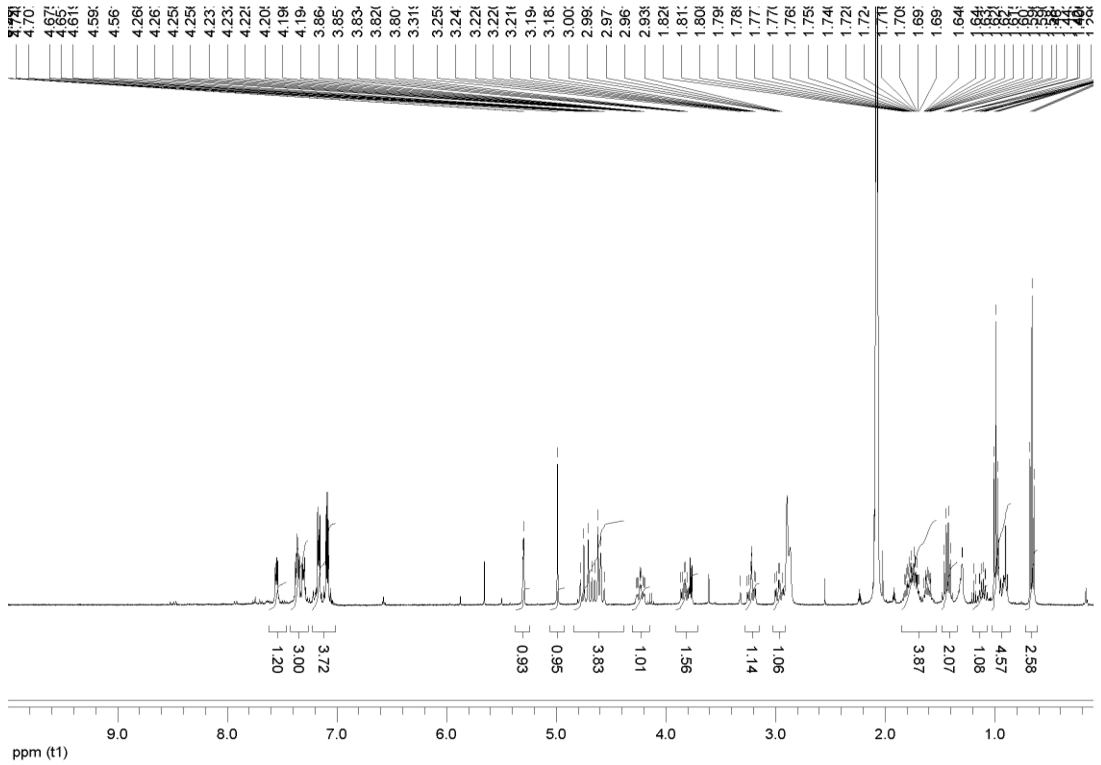

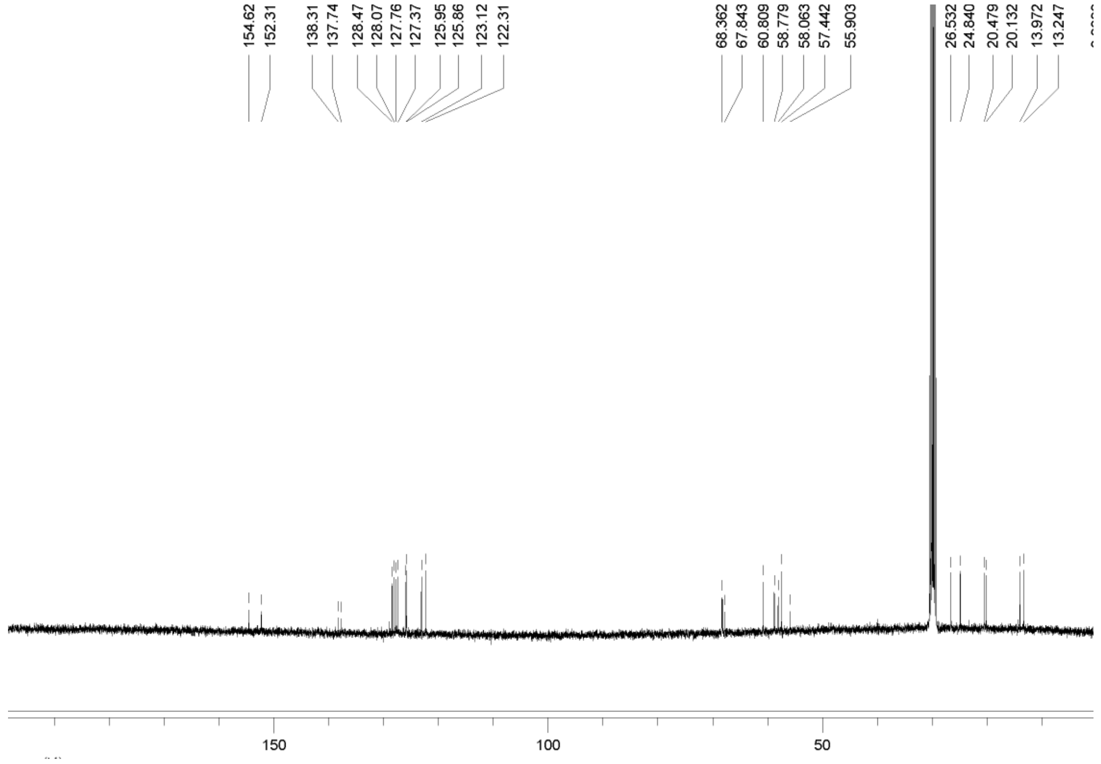

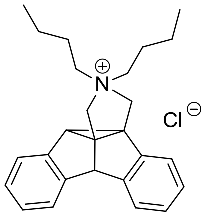

rac-**3d**

in CD_3OD

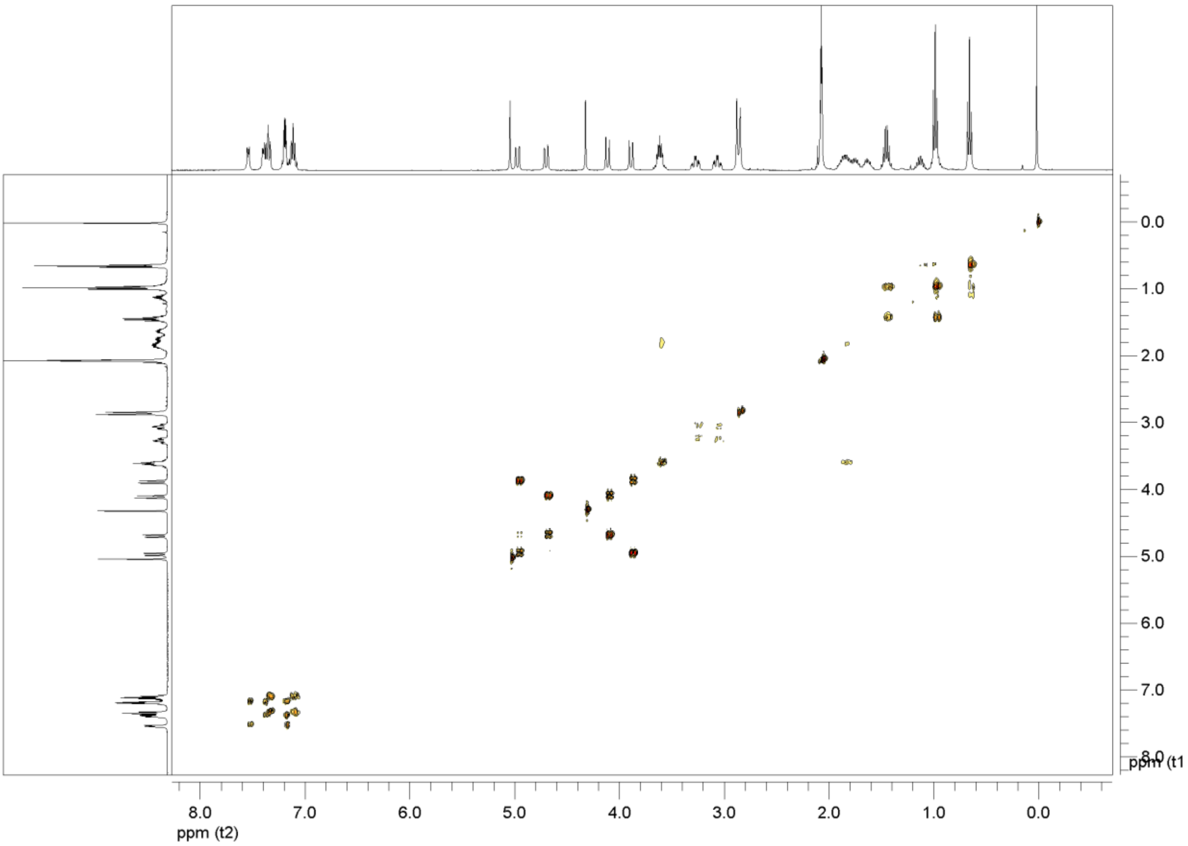

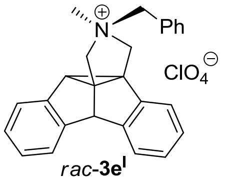

rac-**3e**[I]

in $(CD_3)_2CO$

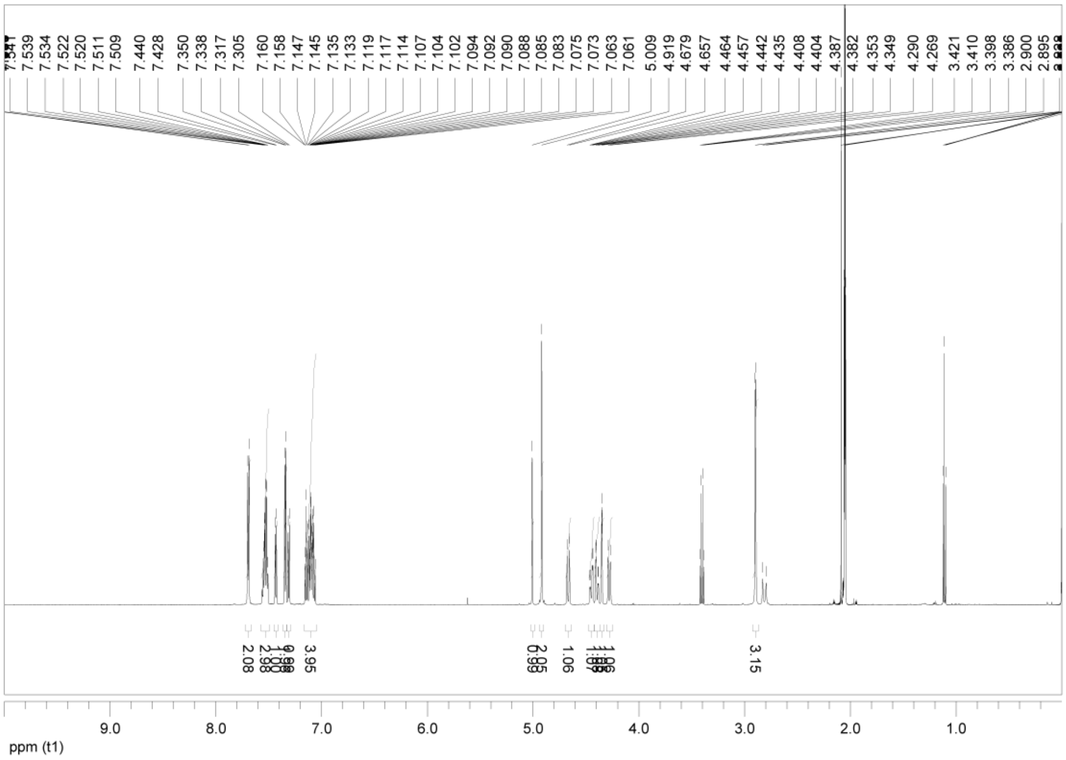

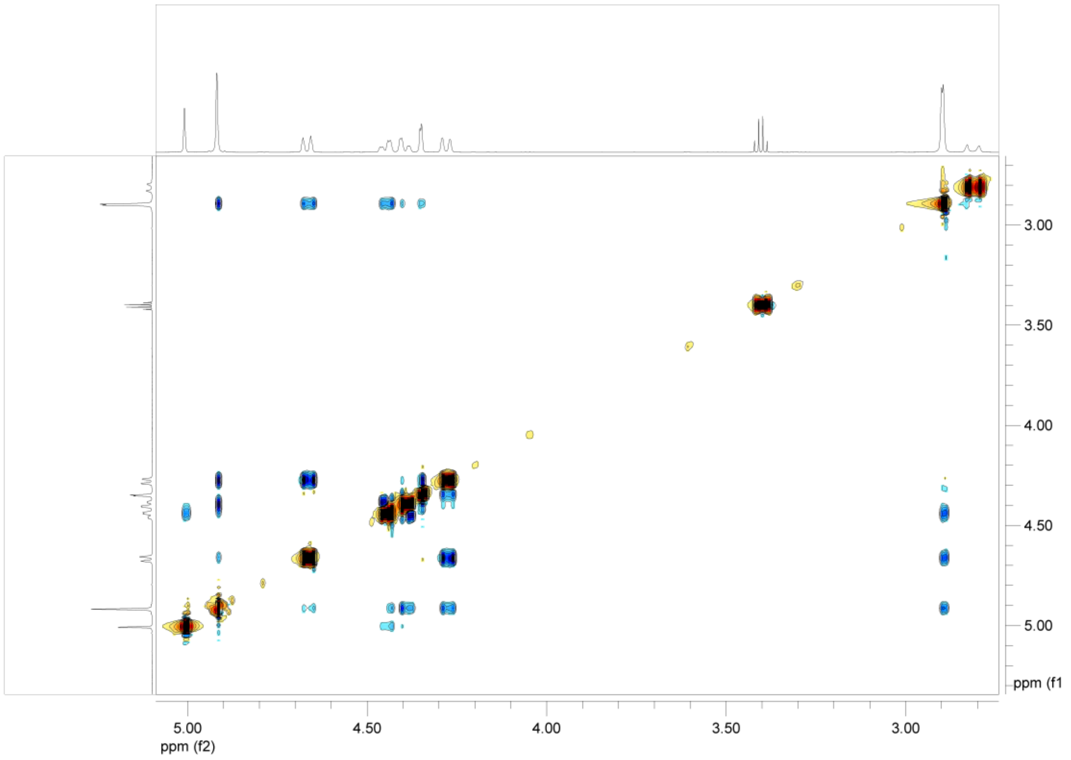

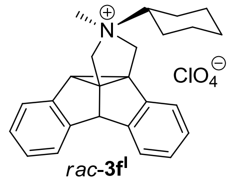

rac-**3f**[I]

in $(CD_3)_2CO$

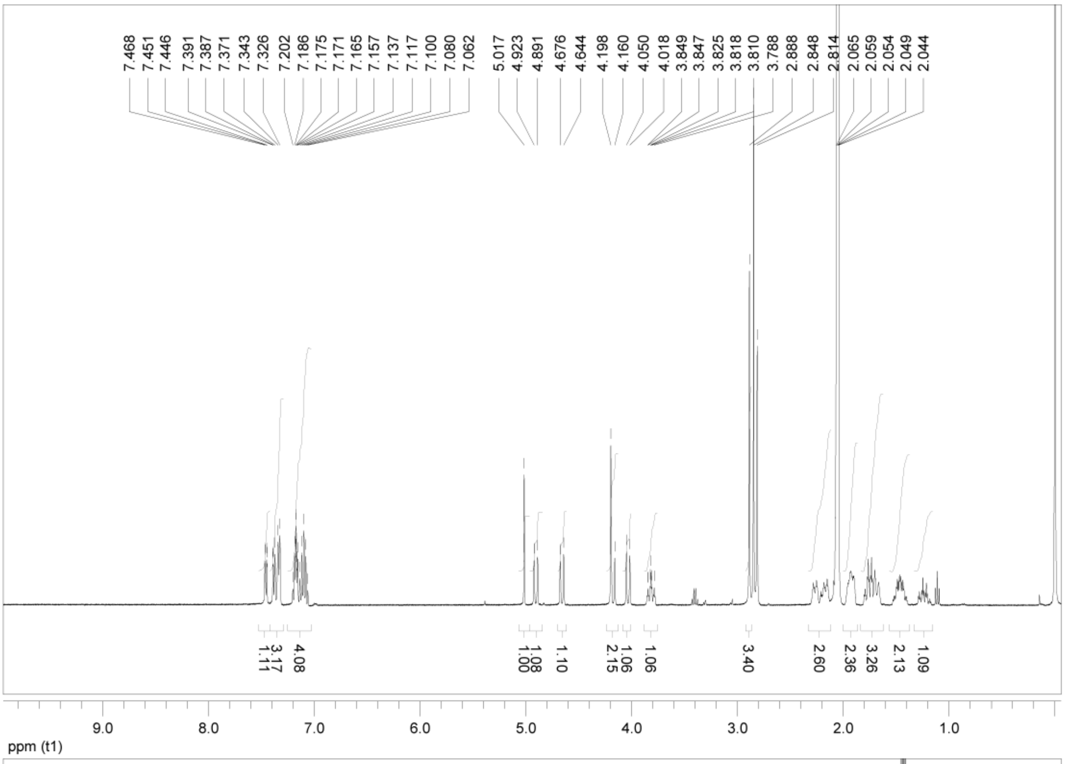

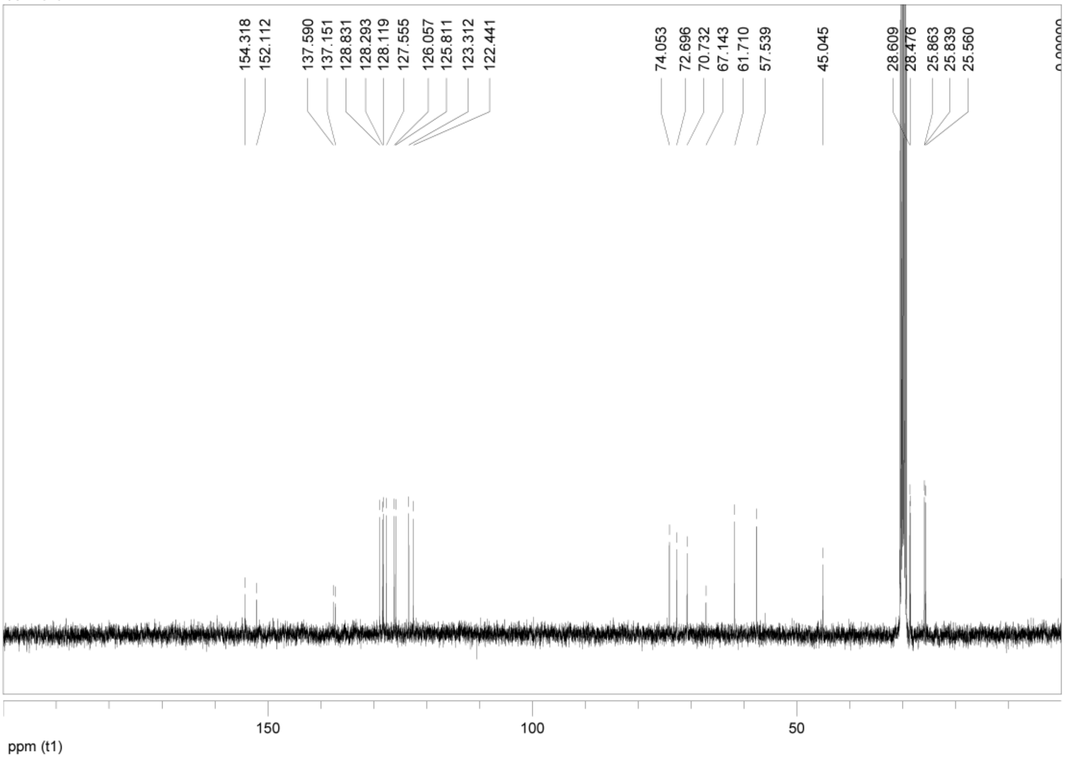

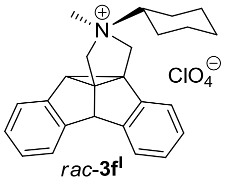

rac-**3f**[l]

in $(CD_3)_2CO$

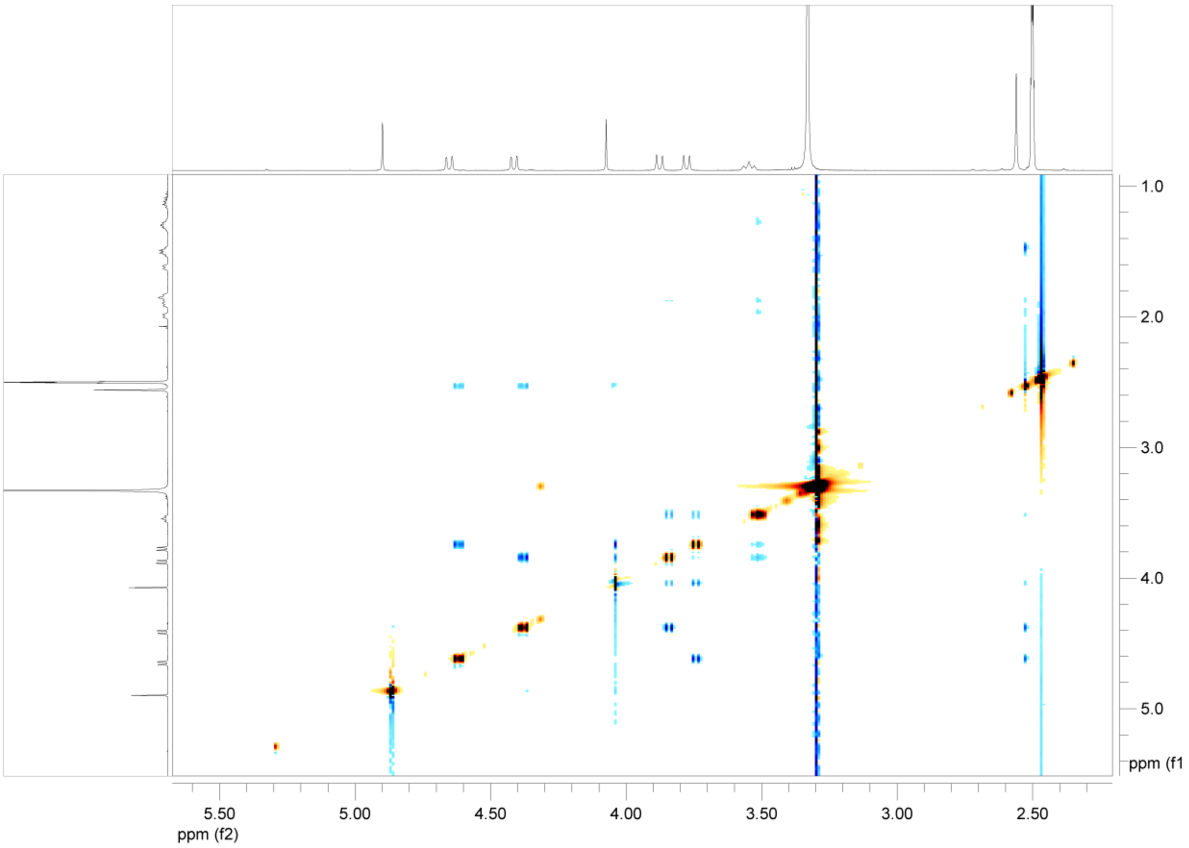

Supplement: File 1 — Experimental procedures, characterization data and copies of 1H NMR and 13C NMR spectra of compounds 2a–g and 3a–f. [file Beilstein_J_Org_Chem-07-119-s001.pdf]
